# Imperceptible augmentation of living systems with organic bioelectronic fibres

**DOI:** 10.1038/s41928-024-01174-4

**Published:** 2024-05-24

**Authors:** Wenyu Wang, Yifei Pan, Yuan Shui, Tawfique Hasan, Iek Man Lei, Stanley Gong Sheng Ka, Thierry Savin, Santiago Velasco-Bosom, Yang Cao, Susannah B. P. McLaren, Yuze Cao, Fengzhu Xiong, George G. Malliaras, Yan Yan Shery Huang

**Affiliations:** 1 Department of Engineering, University of Cambridge, Cambridge, UK; 2 The Nanoscience Centre, University of Cambridge, Cambridge, UK; 3 Cambridge Graphene Centre, University of Cambridge, Cambridge, UK; 4 Department of Electromechanical Engineering, University of Macau, Macao, China; 5 Wellcome Trust/CRUK Gurdon Institute, University of Cambridge, Cambridge, UK; 6 Department of Physiology, Development and Neuroscience, University of Cambridge, Cambridge, UK

**Keywords:** Electrical and electronic engineering, Biomedical engineering, Materials for devices

## Abstract

The functional and sensory augmentation of living structures, such as human skin and plant epidermis, with electronics can be used to create platforms for health management and environmental monitoring. Ideally, such bioelectronic interfaces should not obstruct the inherent sensations and physiological changes of their hosts. The full life cycle of the interfaces should also be designed to minimize their environmental footprint. Here we report imperceptible augmentation of living systems through in situ tethering of organic bioelectronic fibres. Using an orbital spinning technique, substrate-free and open fibre networks—which are based on poly (3,4-ethylenedioxythiophene):polystyrene sulfonate—can be tethered to biological surfaces, including fingertips, chick embryos and plants. We use customizable fibre networks to create on-skin electrodes that can record electrocardiogram and electromyography signals, skin-gated organic electrochemical transistors and augmented touch and plant interfaces. We also show that the fibres can be used to couple prefabricated microelectronics and electronic textiles, and that the fibres can be repaired, upgraded and recycled.

## Abstract (web summary)

With the help of an orbital spinning technique, substrate-free open networks of imperceptible fibres can be created on a range of biological surfaces, providing on-skin sensors that can record electrocardiogram signals, skin-gated organic electrochemical transistors, and augmented touch and plant interfaces.

## Main

Merging biological systems with electronic devices could transform the way we interact and perceive our surroundings^1–9^, providing, for example, data collection platforms for health management and environmental monitoring^10–16^. One goal in the development of functional and perceptual augmentation is to provide intimate bioelectronic device integration with living structures while minimally perturbing the biological functions of the host. Thin-film technologies^14,16^ can be used to create flexible electronics that conform to the macroscopic shape of biological surfaces, but their plastic substrates (around 3–10 μm thick) limit moisture and gas permeability. Electronic textiles^6,17^ use fibre materials or fibre-shaped devices and can offer enhanced comfort and breathability, but existing electronic textile fibre sizes are typically in the range of hundreds of micrometres, prohibiting intimate bio-integration.

Recent advances in stretchable electronics^4,14,18^, electronic skins^1,10,19^, nanomembranes^3,4,20^ and nanomesh structures^2,10,21^ have led to augmentation technologies that are gas permeable^2,3,10,20,21^ and mechanically imperceptible to human skin^1,10,19^. However, such a level of imperceptibility is still a challenge when faced with multi-faceted surface and bulk functions of living structures^22,23^. In particular, biological pores, sensory receptors and topography features^22^ can be concealed when films or components with limited openness are attached over large areas. Furthermore, the pressure exertion that is needed to transfer and deploy premade devices can preclude their use on deformation-sensitive surfaces.

The development of augmented living systems also needs to consider issues related to sustainability. Lithography-based microfabrication is energy and waste intensive due to the toxic chemicals used, the need for sacrificial templates and the effort involved in maintaining clean environments^24^. The production and processing of traditional fibres and textiles also has large carbon and water footprints^25–27^. In addition, the functionalization of living structures that undergo dynamic transformations, or interact with biological analytes or chemical pollutants, can require regular full-scale renewal of the augmentation devices, which is environmentally costly.

**Fig. 1:**
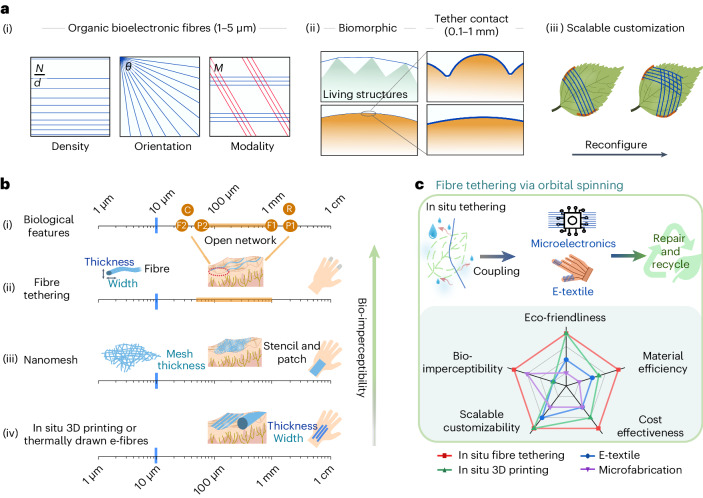
Imperceptibly augmented living structures with organic bioelectronic fibres. **a**, (i) Fibre number density (number of fibres (*N*) over the width of the fibre array (*d*)), fibre orientation (*θ*) and modalities (fibre materials and pattern design) can be customized during in situ fibre tethering; (ii) intimate contacts are achieved between the organic bioelectronic fibres (each around 1–5 µm wide) and different biological surfaces with micro- to millimetre-scaled topographies and (iii) the bioelectronic fibre arrays are reconfigurable to support scalable customization of electronic and sensing elements on living structures in situ. **b**, Length scales and/or feature sizes are indicated for (i) biological structures on the human skin of a hand, including sweat pores (density around 250–500 per cm^2^, symbol P1; pore size around 60–80 µm, symbol P2, ref. ^55^), fingerprint ridges (millimetre ridge-to-ridge spacing, symbol F1 and ridge height of around 20–40 µm, symbol F2, ref. ^56^), single skin cells (sizes around 30 µm, symbol C^57^) and receptor fields on the fingertip (around the millimetre range, symbol R^58^); (ii) bioelectronic fibre tethering for its fibre width, thickness and network opening; (iii) nanomesh for its mesh thickness and mesh opening^2,5,21^ and (iv) in situ printing or thermally drawn e-fibres for their line (or fibre) width and thickness^6,31^. A network or mesh of fibres is considered as fully skin imperceptible if it simultaneously fulfils the conditions of: (1) network or mesh opening between fibres greater than around 50 µm (the sweat gland pore size), but smaller than 1 mm (the fingertip receptor field); (2) width of individual fibres and thickness of the network or mesh smaller than around 10 µm (such that individual skin cells are mostly exposed through the open fibre network, and the fingerprint ridge features are not compromised). **c**, In situ fibre tethering can be used to couple prefabricated microelectronics and electronic textiles, while supporting on-demand device repair, upgrade and recycle. Multi-faceted key performance indicators are compared for different methods for fabricating fibre-like building blocks, where the scales of 1–4 are assigned as 4 = excellent, 3 = very good, 2 = acceptable and 1 = needing improvement. The scores are assigned considering the literature^4–6,9,10,13,17,18,30,31,37^, and discussion in Supplementary Notes 7 and 8 and Supplementary Table 1.

Spiders build sophisticated fibre networks in situ, which are adapted to the environments and require minimal material consumption. Taking inspiration from spider webs, living structures could be augmented with bioelectronics based on designable open network architectures that use individual microscale fibres as building blocks. Such networks could be tethered onto living structures with tuneable fibre number density, orientation and modalities (Fig. 1a). Three-dimensional (3D) printing is considered an environmentally friendly fabrication route^28^ that offers on-demand fabrication^29–32^. However, the resolution of state-of-the-art in situ printing is limited to hundreds of micrometres^30,31^, which compromises device imperceptibility at the biological interfaces. On the other hand, existing approaches for fibre production—such as wet spinning^33^, melting spinning^34^ or electrospinning^35^—can produce micro- and nano-fibres on a large scale, but lack advanced bioelectronic functions. Due to the low bending stiffness and low aerial footprint for surface adhesion of micro-scaled fibres, prefunctionalized fibre networks with open architectures are difficult to manipulate, and cannot be readily transferred and attached onto target objects^36^. In situ generation of fibrous scaffolds is possible^5,37^, but existing techniques result in micro- and nanomeshes with random fibre overlays, and lack control in terms of fibrous patterns, surface contacts and mesoscale network openness (Fig. 1b and Supplementary Table 1).

In this Article, we report the imperceptible augmentation of living structures through the in situ solution fibre tethering of poly (3,4-ethylenedioxythiophene):polystyrene sulfonate (PEDOT:PSS)-based organic bioelectronic fibres. Our approach creates fibre interfaces that can be upgraded and repaired, and requires low material use and generate minimal waste (Fig. 1c). Our strategy could also extend the service duration of disposable and reusable components, enhancing supply-chain resilience.

## In situ tethering of organic bioelectronic fibre

**Fig. 2:**
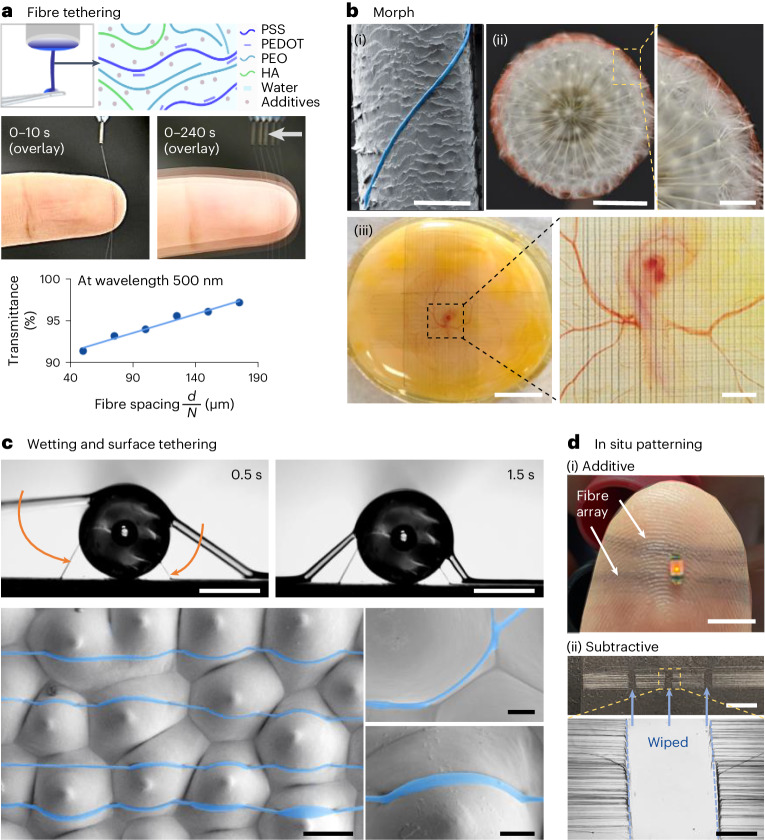
Organic bioelectronic fibre fabrication, morphing and tethering. **a**, Fibre tethering process, where a fibre is first initiated from an aqueous viscoelastic solution, and then drawn to tether around the target object. An example fibre deposition process is shown on a fingertip. The bioelectronic fibre array transmittance shows a linear relationship with fibre spacing $\frac{d}{N}$, with the best linear fitting of *T*(%) = 0.045 $\frac{d}{N}$ + 89. **b**, Photographs and scanning electron microscopy images showing the fibre morphing morpholog**i**es, for (i) a fibre (with a false colour highlight) on a human hair (scale bar is 50 μm); (ii) fibres with a red colour dye conform on a dandelion seedhead, and a zoom-in view (scale bars left to right, 1 cm, 1 mm) and (iii) fibre grids on a day-3 chicken embryo in a petri dish, and a zoom-in view (with fibres deposited on top of the vitelline membrane covering the yolk; scale bars left to right 5 mm, 500 μm). **c**, Fibre-surface tethering and wetting on a glass rod (light microscopy time lapse photographs), and an orchid flower petal (scanning electron microscopy images, with fibres highlighted with a false blue colour) (scale bars left to right and top to bottom, 500, 500, 50, 10 and 10 μm). **d**, Concepts for fibre patterning, through (i) additive (fibre deposition) and (ii) subtractive (fibre erasing) processes (scale bars top to bottom, 5 mm, 5 mm and 500 μm). Source data

Our organic bioelectronic fibres are based on PEDOT:PSS (a mixed ionic and electronic conducting polymer that have proven in vivo biocompatibility^38^), hyaluronic acid (a skin extracellular matrix analogue^39^ that helps fibre spinnability and skin contact) and polyethylene oxide. The bioelectronic fibres are produced from a solution phase at ambient conditions, where the solution spinnability was characterized by the ratio of shear modulus over surface tension (Supplementary Fig. 7). We design an orbital spinning approach to control the bioelectronic fibre tethering and patterning directly onto living structures. Using the target periphery as a template, the fibre tethering is physically guided by the shape and position of the target object (Supplementary Fig. 8). Aided by the dynamic physically intelligent morphing mechanism of fibre tethering, in situ construction of fibre interface over a centimetre-sized target (such as a person’s finger) does not require digital replica (Supplementary Video 1). In addition, the tethering process is tolerant to target movements for an electrode-patch application (Fig. 2a). Each rotating arm orbit results in one strand of solution fibre to be drawn onto the target. The fibre deposition path planning allows direct formation of fibre-to-surface and fibre-to-electrical contact connection (Supplementary Fig. 9). Thus, the entire fibre deposition process is contactless and mask-free. Controlling the fibre number density—the number of fibres (*N*) over the width of the fibre array (*d*), or *N*/*d*—allows tuning of the bulk optical property of the bioelectronic fibre patterns from transparent (transmittance of around 98%, for a *N*/*d* around five per mm) to semitransparent (transmittance of around 91%, for *N*/*d* around 20 per millimetre) (Fig. 2a and Supplementary Fig. 10). A summary of the bioelectronic fibre number densities used in various applications with the relationship to network opening and transparency is provided in Supplementary Table 2. The solution feeding consumes a total of around 1 µl of solution per minute for direct fibre deposition. Considering typical fibre networks created within 2–5 minutes, the total solution usage is around 2 to 5 µl and the total embodied dry mass input could be estimated as 0.1–0.3 mg per fibre network device (Supplementary Note 1).

The fibre tethering process is suitable for a range of diverse biological objects with curved and irregular surfaces, from the width of a human hair, to ridges of a fingertip and chick embryos (Fig. 2b and Supplementary Fig. 11a,b). The bioelectronic fibre tethering process induces little perturbance to the targets’ surface structures, where the force of a single fibre tethering is estimated to be in the range of 10 μN via cantilever experiments (Supplementary Note 2). We show that *Mimosa pudica*, a touch sensitive plant^40^ that closes on gentle hand touch with a force of around 200 μN, does not respond to the fibre deposition process (Supplementary Video 2). The mechanical effects of fibre tethering on biocompatibility was further evaluated using fragile day-2 chicken embryos, whose development is highly sensitive to external forces and stresses^41^. Our results show that the day-2 chicken embryos with fibre networks on the developing tissue display normal growth rates and morphological changes through 24 hours postfibre tethering (Supplementary Video 3 and Supplementary Fig. 11c).

The fibres are spun in a solution and/or wet state, meaning that abundant residual water remains in the ‘wet fibre’ upon surface tethering (Supplementary Notes 3 and 4); which leads to a dominant Wenzel-like fibre-surface contact state. Furthermore, experimental results and theoretical analysis indicate that under the current formulation and spinning settings, intimate contacts on convex and solid structures over hundreds of micrometres and various topographical features are expected to form (Supplementary note 4 and Supplementary Video 4). As shown in Fig. 2c, the bioelectronic fibre forms dominant intimate attachments even down to the micrometre-level surface topographies for macroscopically convex surfaces (in contrast to non-contacting fibres produced by control solutions, Supplementary Fig. 12). Thus, the average feature size of a single bioelectronic fibre ranges between 1 and 5 µm depending on the contact states of different surfaces (Supplementary Fig. 13). The spatially patterned bioelectronic fibres, along with their mechanical erasability in a wet state (as shown by fibre mechanical characterization in Supplementary Note 5), offer possibilities to create in situ patterning through both ‘additive’ and ‘subtractive’ modes (Fig. 2d and further results later).

## Performance tailoring of on-skin electrodes

**Fig. 3:**
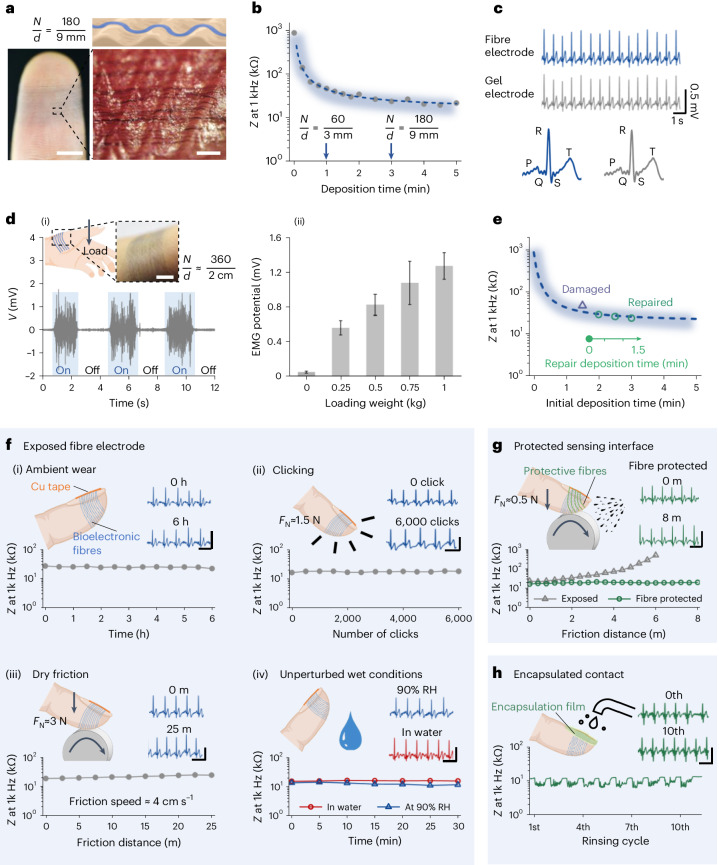
Tailored imperceptible on-skin electrodes. **a**, An illustration (top) showing fibres covering the ridges of the fingerprints, where $\frac{N}{d}$ indicates the number of fibres *N* across a distance *d*; and the experimental evidence is provided by the photographs (bottom row) showing the complete fibre array on a fingertip and the zoom-in view of the fibres follow the ridges of the fingerprints (scale bars left to right, 5 mm, 500 μm). **b**, Contact impedance versus deposition time on the fingertip. **c**, Comparison of ECG signals acquired by fibre and gel electrodes at the same time (signal correlation *P* = 0.99). **d**, (i) An array of fibres deposited on the thumb muscle region, where ON/OFF loading on the thumb results in clear on/off EMG signals detected by the fibres (scale bar, 1 cm). (ii) Bar chart to depict variations in absolute EMG amplitude from the thumb muscle region against different loading weights on the thumb (data are presented as mean absolute EMG values ± standard deviation of EMG measured for around 5 s in each case). **e**, Facile repairability of the exposed fibre arrays. The triangular symbol indicates the impedance of the fibre arrays after being deliberately damaged by abrasion, and then new fibres are deposited on demand to repair as indicated by the circular symbols. **f**, The stability of exposed fibre electrode (exposed bioelectronic fibres on skin) under the conditions of (i) ambient wearing; (ii) mouse clicking; (iii) dry friction wear with a plastic surface (at a surface speed of 4 cm s^−1^ under around 40% relative humidity (RH) environments); (iv) simulated ‘wet’ conditions without mechanical disturbance. **g**, Wet friction (at a surface speed of around 4 cm s^−1^) of exposed and cellulose-based fibre protected sensing interface. **h**, Rinsing under running water (the sensing interface is protected with cellulose-based fibres and the fibre contact is encapsulated with a cellulose-based film) (ECG scales for **f**,**g**, horizontal time scale 1 s, vertical voltage scale 0.5 mV). (**a**–**e**, typical results from *n* = 5 volunteers, **f**–**h**, typical results from *n* = 3 volunteers, for all experiments with *n* > 3 independent experiments performed on each volunteer). Source data

A fresh fibre electrode on a fingertip (Fig. 3a), with contact impedance comparable to reported microfabricated gold nanomeshes^21^, can be created within 3 minutes of fibre tethering using the current single nozzle setting (Fig. 3b and Supplementary Fig. 14a,b). The interfacial contact impedance of the fabricated fibre electrodes falls within the range of around 20–40 kΩ at 1 kHz (Supplementary Fig. 14c). Such consistency in deploying the fibre electrodes indicates that the functions of the fibre patch are negligibly affected by positional drifts of the target during in situ fibre tethering. Figure 3c and Supplementary Fig. 15 show that electrocardiogram (ECG) signals acquired by the bioelectronic fibre array are consistent with the ECG signals collected by a reference gel electrode at the same time (Supplementary Video 5). Similarly, the fibre arrays can be configured to acquire electromyography (EMG) signals, and to monitor the steady increase of EMG signal amplitudes as representing the increased electrical activities of the skeleton muscles due to external loadings (Fig. 3d and Supplementary Fig. 16).

Repairability is a potential advantage of tethering the organic bioelectronic fibres as an exposed transient electrode. Deliberately damaging exposed fibres results in the fibre electrode–skin contact impedance at 1 kHz to increase from around 20 to around 50 kΩ, which affects the ECG sensing performance (Fig. 3e and Supplementary Fig. 14e). New fibres were deposited on demand to repair the fibre electrode without affecting existing interconnections. The biopotential acquisition interface was fully renewed to recover the original contact impedance level and ECG sensing performance with a fraction of material inputs compared to creating a new electrode.

Next, we show that the device and contact formats of the bioelectronic fibres on a fingertip can be customized to withstand environmental and ‘touch’ perturbations simulating daily fingertip experiences (Supplementary Table 1). The tethered bioelectronic fibres, even in their fully exposed states, show stable electromechanical performance under various dry wearing conditions and environmental disturbances such as water-soaking, humid and mild heat (Fig. 3f). The specific conditions tested include: (1) ambient wear for at least 6 hours; (2) clicking a mouse for more than 6,000 times with a mean clicking force of around 1.5 N; (3) around 25 m of dry frictional wear through contact with a plastic surface with a mean normal force up to 3 N; (4) simulated ‘wet’ or ‘heat’ conditions without mechanical disturbance (such as immersion for at least 30 minutes in water or 90% relative humidity, or under an environment of around 40 °C) (Fig. 3f, Supplementary Fig. 17 and Supplementary Video 6). Under these conditions, the on-skin fibre patterns exhibited no visible macroscopic distortion, and the performance does not degrade by much in terms of interfacial contact impedance and ECG acquisition. The conformally attached bioelectronic fibres form good fibre-to-skin adhesion, with the maximum recorded peeling force approaching around 15 N m^−1^ (Supplementary Fig. 18). We note that the strength, and thus the electromechanical performance of the bioelectronic fibres, is affected by the level of fibre hydration as shown in Supplementary Note 5. Therefore, under wet mechanical disturbances (such as rinsing with water), the exposed bioelectronic fibres on the fingertip become unstable.

Further enhancement in the ‘wet-stability’ of the device interfaces is achievable through the addition of biocompatible and biodegradable cellulose-based materials as protective layers. As a conceptual demonstration cellulose-based fibres can be added on top of the bioelectronic fibres, to improve the overall fibre device’s electromechanical stability (Fig. 3g and Supplementary Fig. 19). With a cellulose-based protective layers, the tethered bioelectronic fibre array can maintain its as-deposited performance for around 8 m of wet friction with a normal force of around 0.5 N, and at least 1 hour of computer typing and office work. Furthermore, stability improvements were made by encapsulating the exposed fibre contact area—the area on the nail in which the connection is made between the bioelectronic fibres and a copper tape—with a cellulose-based film of around 2 μm thickness. In this case, the entire fibre device on the fingertip could withstand at least ten cycles of 30 seconds running water rinsing (Fig. 3h and Supplementary Fig. 20).

## Substrate-free fibres for imperceptible augmentation

**Fig. 4:**
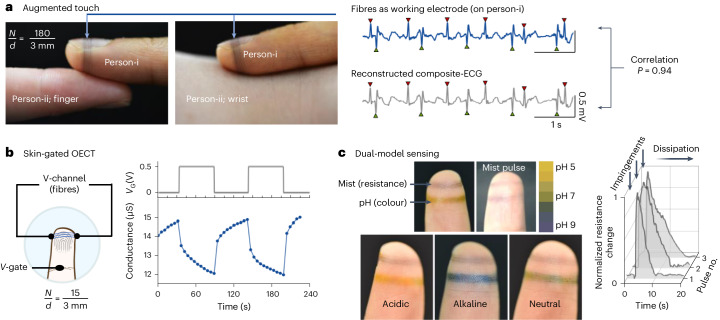
Imperceptible augmentation. **a**, Augmented touch perception via dual-ECG sensing with person-i wearing bioelectronic fibre arrays and person-ii without. The dual-ECG signal acquired through the fibre array is compared with the reconstructed composite-ECG signal from validation gel electrodes. The red downward facing and green upward facing triangles indicate the R peaks of person-i and person-ii, respectively. **b**, A breathable skin-gated OECT on a fingertip; the OECT displays a response time in the 60 s range. **c**, Dual-modal sensing for augmented perception of mist pulses with acidic, alkaline and neutral compositions distinguished through colorimetric and electrical readouts. The mist pulse photographs show an example of a neutral mist pulse, and the fibre resistance change was recorded by applying three consecutive neutral mist pulses (normalized resistance change is calculated as $\frac{{R}^{* }-{R}_{0}}{{R}_{0}}$, where R* is the peak resistance and *R*_0_ is the initial resistance; the initial resistances of the fibre arrays are in the range of 10 kΩ). **a**–**c**, Typical results from *n* = 5 volunteers, with *n* > 3 independent experiments performed on each volunteer. Source data

Substrate-free bioelectronic fibre patterns can be used for various customized augmentation applications. First, as both sides of the bioelectronic fibres can remain exposed when worn on the finger, the wearer (person-i) can detect another individual’s (person-ii’s) ECG by making physical contact between their wearable electrode and the other person’s (person-ii’s) bare finger or wrist (Fig. 4a). The dual-ECG signals acquired by the fibre electrodes contain ECG characteristics of both people: the R peaks of person-i are pointing upwards because the person-i ECG is measured from the left to right hand; while the R peaks of person-ii points downwards because it is measured from the right to left hand (in the reverse direction compared to person-i). The upward and downward R peaks for both people can be separated through signal analysis (Supplementary Fig. 21). We note that the dual-ECG signals measured from the fibre electrodes show a high correlation coefficient (*P* value is 0.94) with the reconstructed composite-ECG signal measured from individual’s validation gel electrodes. In future work, advanced signal processing techniques, including machine learning and blind signal separation^42^, could be used for ECG signal separation and identification of other minor peaks from the dual-ECG signals. Because the fibre arrays are substrate-free, and the open fibre network minimally conceals the skin surfaces, the subtle touch sensations of the volunteers are preserved so that they can simultaneously feel the blood vessel pulsations underneath the skin.

Our fibres can be configured into organic electrochemical transistors (OECT) because of the semiconducting nature of PEDOT:PSS^38^. We fabricated a breathable skin-gated OECT on the fingertip using the tethered bioelectronic fibres, where the area of skin acts as electrolyte between the gate and the substrate-free channel fibre arrays. The conformal contact facilitates charge exchange at the skin–fibre interface, and this enables the gating of our OECT using skin as the gate-channel electrolyte (Fig. 4b and Supplementary Note 6). A positive current flows through the fibre array when a channel voltage is applied (at 30 s), then the current drops abruptly as expected for PEDOT:PSS channel material operating in depletion mode (a positive gate voltage switches off the device, and vice versa). The removal of the gate voltage at 90 s leads to the recovery of the current (hence the recovery of the channel conductance). Repeated gate voltage pulses result in similar current responses, showing the fibre array remains structurally intact during the switching processes.

Furthermore, complementing the bioelectronic fibres with other fibres possessing different sensing modalities offers the opportunity to create multi-modal sensors at the same anatomical site. Humans do not possess skin ‘wetness’ receptors, and ‘wetness’ is interpreted individually through perceptions of temperature and mechanical inputs^43^. The resistance of PEDOT:PSS materials is moisture dependent^44^. As an intuitive illustration, Supplementary Video 6 shows that bioelectronic fibres tethered on a dandelion seedhead can be used to detect the environmental moisture flow without concealing the seedhead’s fine hairs. As a further conceptual demonstration of imperceptible augmented mist pulse perception, bioelectronic fibres and colorimetric pH-responsive fibres were both looped on the index finger of a person (Fig. 4c, see Methods for the fabrication of pH-responsive fibres). The temporal resistance of bioelectronic fibres increases on emersion in water mist pulses. Impingement of acidic, neutral or alkaline mist pulses onto the finger can be distinguished by simultaneously monitoring the bioelectronic fibres’ temporal resistance and the pH-responsive fibres’ colour. Dual-modal sensing (mist detection by bioelectronic fibres, and pH by colorimetric fibres) was used here. When similar pH mists repeatedly impinge on the fingertip, the mist flows can be detected by the changes in electrical resistance of the bioelectronic fibres, but the colorimetric fibres’ colour remains the same. Because the fibre arrays are substrate-free and minimally conceal the skin surfaces, all volunteers were able to still feel the subtle sensations generated by the mist flow impinging on the fibre arrays.

## Adaptive and reconfigurable fibre sensing arrays and networks

**Fig. 5:**
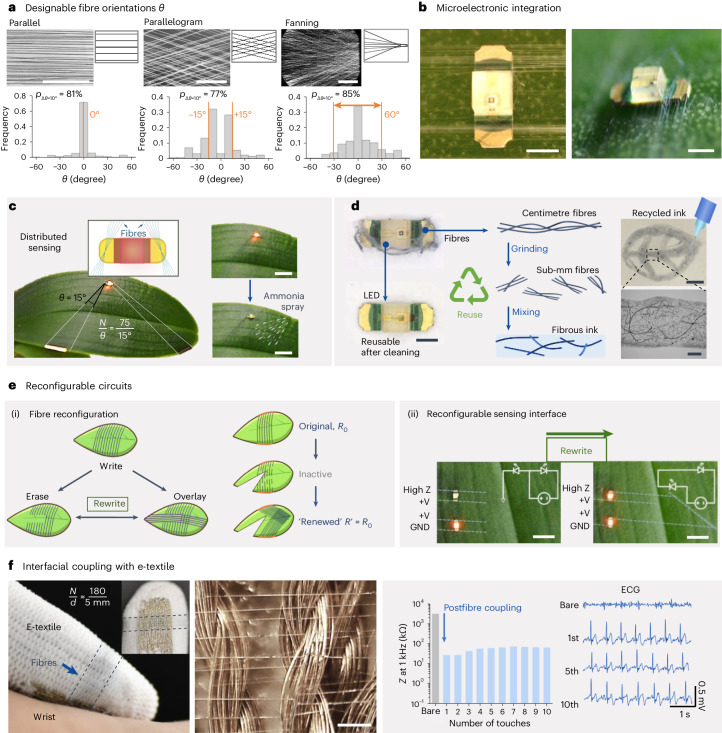
Adaptable, versatile and reconfigurable fibre coupling. **a**, Fibre tethering with designable fibre orientations (*θ*) is demonstrated by statistical analysis of fibre orientations *θ* with different fibre patterns, and the patterning accuracy (*p*_*∆θ*=10°_) is calculated by taking an 10°-offset (the width of the binning in the histogram) being an acceptable criterion for misalignment (a horizontal line is used as the 0° baseline for measuring all the fibre orientation angles). **b**, Photographs showing the top and profile views of fibre arrays connecting to the contacts of an LED (scale bars, 2 mm). **c**, Distributed bioelectronic fibres to connect with an LED on a plant leaf to warn environmental exposure of ammonia on the plant surfaces (where the dashed lines indicate the boundary of the fibre arrays, scale bars, 5 mm; typical results from independent experiments performed with *n* > 3 plants). **d**, Concepts for reusable and recyclable components, where the LED and bioelectronic fibres could be separated: the LED reused, and the fibres recycled into a feedstock to create conductive inks for 3D printing (line resistance at around 1 kΩ mm^−1^ dependent on filler concentration; scale bars from left to right, and top to bottom, 1 mm, 2 mm and 500 μm). **e**, Concepts for reconfigurable sensing interface. (ii) Schematics showing a fibre fabrication and reconfiguration process, where the ‘rewrite’ process could ‘renew’ the fibre sensing interface, while retaining similar levels of fibre array resistance between the original *R*_0_ and the ‘renewed’ *R*′ states and (ii) bioelectronic fibre arrays on the surface of a leaf that are written and rewritten to achieve a topological change in the sensing interface (for each fibre array $\frac{N}{d} \approx \frac{60}{1\,{\mathrm{mm}}}$; scale bars, 5 mm; typical results from independent experiments performed *n* > 3 plants). **f**, An array of bioelectronic fibres deposited onto the finger region of an e-textile glove that reduce contact impedance by approximately two orders of magnitude, thus enabling biopotential monitoring (scale bar, 200 μm). Source data

We further demonstrate adaptive and reconfigurable sensing systems based on our bioelectronic fibres (as the sensing elements), which could be coupled with prefabricated microelectronics or e-textile wearables. The ability to control the fibre orientations (*θ*) enable versatile interconnections to be made for devices. We demonstrate parallel (*θ* = 0°), parallelogram (*θ* = ±15°) and fanning (−30° < *θ* < 30°) patterns. The estimated patterning precisions were above around 75% (Fig. 5a). Our patterning precision estimations are affected by process-intrinsic factors of the orbital spinning, including mechanical controls and environmental disturbances (such as wind) during patterning, and is also affected by postdata analysis issues such as mis-identification due to automatic image registration of the fibres. Individual fibres’ continuity and form factor thus support the connection of small electronic devices such as a micro-light emitting diode (LED) without adhesives (Fig. 5b). The low deposition forces imposed by the orbital spinning and fibre tethering (Supplementary Note 2) mean that the electronic device, such as a micro-LED used here with a weight of around 1.4 × 10^−5^ N, stays still during the circuit formation because of gravity and the friction of the living structure, such as leaf texture. Thus, considering the failure force per fibre during debonding was measured to be around 3.5 × 10^−5^ N (Supplementary Fig. 17), as few as a single fibre is sufficient to support the weight of one micro-LED. Cyclic voltammetry through the bioelectronic fibres showed ohmic behaviour for applied voltage per distance under 6 V cm^−1^ (Supplementary Fig. 23), and thus our fibres are compatible with other low-power bio-safe electronic components.

We showed that on a plant our distributed bioelectronic fibres can be used to connect a micro-LED and can be used as a display to form a warning system for elevated levels of ammonia exposure (Fig. 5c). Ammonia is a type of n-type dopant that interacts with PEDOT:PSS in the bioelectronic fibres to cause a dedoping of the PEDOT:PSS polymer backbone^45^; hence, the micro-LED dims non-reversibly on ammonia exposure (Supplementary Fig. 22a). In comparison, the micro-LED light only dims temporarily when exposed to water mist because the effect of water on the resistance of PEDOT:PSS is reversible (Supplementary Fig. 22b and Supplementary Video 7). The designed bioelectronic fibre pattern widens the ammonia mist capture area without compromising breathability and light transmission for photosynthesis of the leaf surface (over 90% transmittance was measured, see Fig. 2a). The fibre array, which acts as a transient interface, can be renewed independent of the other discrete electronic components such as a reusable LED. After mechanical separation from the leaf our fibre arrays can be recycled, through grinding and sonication to produce a conducting fibre-loaded ink for 3D printing (Fig. 5d).

We show a rewritable and reconfigurable fibre array and network on a leaf of a whole plant (Fig. 5e). The fibre tethering enables a fabrication closed-loop of writing, erasing, overlaying (Fig. 5e–i) for in situ sensing interface reconfiguration and renewal. Writing is an additive process that involves deploying fibres in the target area; erasing involves selectively removing fibres, where the weakened strength of bioelectronic fibres in wet regions enables them to be selectively erased off on demand without needing organic solvents and overlay (as an additive process) deploys fibres over existing structures with an arbitrary alignment. When reconfiguring the fibre path for chemiresistive sensing, customizing the number of fibres (*N*) in the array leads to a tuneable circuitry level fibre array resistance (Supplementary Fig. 24). This conceptual reconfigurable sensing interface is advantageous in cases where the leaf surface is damaged or obstructed. The bioelectronic fibres can be renewed by rerouting the fibre path onto the original electrical contact connections without further perturbing the living structure (i.e. the leaf) (Supplementary Fig. 24). Figure 5e(ii) shows that such mask-free direct patterning supports in situ sensing interface repair and reconstruction on living structures with minimal disruption and infinitesimal material usage.

Finally, we demonstrate interface compatibility between the fibre tethering strategy and e-textile wearables (Fig. 5f). Here, bioelectronic fibres were tethered directly onto a glove sewn with metallic conductive yarns. Such tethering provides a dry interfacial coupling that drastically decreases the contact impedance between the metallic yarn of the glove and human skins, enabling biopotential sensing through touch. Afterwards, the bioelectronic fibres, which are coupled to the e-textile through a dry mechanical interface, could be removed from the glove by dry scratching. The collected bioelectronic fibres were recycled as a 3D printing ink (Fig. 5d). Thus, our bioelectronic fibre tethering-enabled augmentation can be considered a sustainable bridging technology, as it offers the possibility to decouple the service durations of disposable, and multi-use, quasi-permanent components.

## Conclusions

We have reported the augmentation of living structures through the in situ tethering of organic bioelectronic fibres. The imperceptible fibres are fabricated on demand and can adapt to the living structures without influencing their biological functions and transformations. By harnessing the viscoelasticity and surface wetting properties of the predry solution during in situ fibre tethering, we created sensing interfaces across biological curvatures and topographies of different scales (such as fingertips and finger ridges). Our in situ fibre tethering approach overcomes materials and format limitations associated with prefabricated interfaces. The fibre tethering process lowers the requirements for intrinsic material stretchability and complex print path planning, while enabling rigid-to-flexible device coupling (such as coupling microelectronics to plant leaves).

Currently, the stretchability of the bioelectronic fibres is limited by the intrinsic material properties of the PEDOT:PSS and polyethylene glycol (PEO). Nevertheless, by modifying the fibre network design patterns and orientations with respect to the stretching direction, the device interface cyclic stretchability can be enhanced to around 15% (Supplementary Fig. 25). In the future, the stretchability of the fibres could be enhanced further by combining with elastomers. Furthermore, the functionality and stability of the resulting biointerfaces could be tailored by mixing and matching a wide range of fibre materials (or fibre modalities).

Today, developments in electronics and sensors need to be focused on more than just increasing device performance, they also need to consider ways to reduce environmental effects over the full life cycle of the devices^24,46–48^. The raw materials used to fabricate our organic bioelectronic fibres, and their assembled device interfaces, are based on earth-abundant and biocompatible materials (including organic semiconductors PEDOT:PSS and cellulose derivatives), and are not reliant on precious metals or supply-chain sensitive sources. Our fibre tethering approach also offers an individually adaptive fabrication strategy with low energy consumption (Supplementary Note 7). The 0.1–0.3 mg of dry mass input that is required to form our fibre networks for each device is equivalent to the estimated microfibre mass released from 1 g of synthetic fabric after machine washing^49^. A typical machine washing cycle of 5 kg of fabric generates a greater environmental cost in terms of water consumption and microparticle production than fabricating 5,000 devices of bioelectronic fibre arrays (Supplementary Note 8). Our material and process strategy for bioelectronic fibre tethering—which includes creation, repair, reconfiguration and recycle steps—thus offers a low material and low energy consumption approach to augmenting living systems with minimal environmental impact.

## Methods

### Solution preparation

Bioelectronic fibres were prepared by mixing a PEDOT:PSS (poly (3,4-ethylenedioxythiophene): polystyrene sulfonate) solution and a PEO and sodium hyaluronate solution. The PEDOT:PSS solution was prepared according to the literature to achieve good conductivity and stability^50^. Here, 95% (v/v) of PEDOT:PSS solution (Heraeus Clevios PH 1000, at around 1% (w/w) aqueous dispersion) was mixed with 5% (v/v) of ethylene glycol (Sigma-Aldrich), and additional 10 μl of dodecylbenzenesulfonic acid (Sigma-Aldrich) was added to per 10 ml of the solution as a surfactant to prevent aggregation. The solution was then sonicated for 20 min. The PEO–HA solution was prepared by dissolving 2% (w/w) 8 MDa PEO and 0.5% (w/w) sodium hyaluronate (Sigma-Aldrich) in deionized water by mild stirring at room temperatures for 48 h. The choice of PEO was based on our previous fibre printing experience^33^, where we demonstrated that the long molecular chain PEO solutions (4 MDa) could be stretched into thin fibres without the need of electrical field. In this work, we used an even higher molecular weight PEO (8 MDa) along with sodium hyaluronate to further enhance the fibre spinnability and fibre interfacial contacts. Before fibre deposition, the PEDOT:PSS solution and PEO solution were mixed together in a two to one (v/v) ratio for achieving good fibre spinnability with sufficient PEDOT:PSS for sensing applications, and then this was stirred for 12 h at room temperatures to form the final bioelectronic fibre solution.

The solution for forming the protective cellulose-based fibre layers was prepared by dissolving 6% (w/w) of ethyl cellulose and 1.5% (w/w) of 8 MDa PEO (Sigma-Aldrich) in 80% (v/v) ethanol, followed by stirring at room temperature for 12 h. This material concentration was adapted from previous literature^51^, and 8 M Da PEO was used in this work to enhance the fibre spinnability.

In the case of dual-modal sensing on the fingertips, a fully aqueous PEO-based fibre solution was used for skin-compatible pH-sensing. To prepare the PEO-base pH-responsive fibre solution, 6% (v/v) nitrazine yellow (a pH-responsive dye, Sigma-Aldrich, of which concentration allows visible fibre colour change with good spinnability) was added to the PEO-base matrix solution for mild stirring at room temperature for 12 h.

### Fibre tethering process

For the current study, the orbital spinning platform for fibre tethering consisted of a cylindrical fibre spinning zone of up to 15 cm in diameter and 30 cm depth. The rotating arm was powered by a servo motor (Parallax 6 V continuous servo) at the rate of roughly 45–65 rpm. The diameter of the spinning zone could be adjusted depending on the size of the target object, and could be varied from 0.5 to 15 cm. The solution feeding system and the rotating arm formed the fibre tethering platform, and the platform could be mounted on a translational stage, a mini-rover or be handheld. The fibre solution was loaded into a 1 ml syringe that was connected to a 6.3 mm (0.25 inch) long 22-gauge blunt-end stainless-steel needle (Adhesive Dispensing Ltd). The syringe and the needle were placed above the rotating arm in the way that the pendant solution droplet at the tip of the needle would just be scratched by the rotating arm. The fibre deposition path was designed such that in each cycle of fibre tethering, the fibre was tethered onto the target surface (for example, the fingertip), as well as the contact electrode (for example, copper tape on the nail) in a single orbital spinning step. The contact electrodes made of copper tapes then connected to a external measuring instrument.

For laboratory-based experiments, pressurized air (Elveflow OB1 microfluidic flow controller) was connected to the syringe to feed the solution (roughly 40 mbar, flow rate roughly 60 µl min^−1^). The linear movement, controlled by a linear translational stage (Thorlabs, catalogue no. MTS50-Z8), was responsible for creating parallel fibre arrays with various densities and the rotational movement, controlled by a servo motor (20 kg high torque servo, SUNFOUNDER), was responsible for creating fibre arrays with various angles.

### Fibre solution rheological characterizations

The shear rheological properties of various fibre solutions were acquired with a Kinexus KNX2112 controlled stress rheometer at 25 °C. A parallel plate configuration was used with 1 mm gap distance. Fibre solution extension measurements were performed with TriMaster. The extension speed was set at 80 mm s^−1^ and the images were acquired by a PHANTOM VEO-E 310L high-speed camera at 3,000 frames per second.

The surface tension of the solutions was determined through pendant drop shape analysis^52^. Solutions were fed steadily (flow rate 300 μl h^−1^) into the outlet of a stainless-steel needle (18 gauge, the outer diameter is 1.8 mm). The photographs of the droplets were taken just before the droplets were about to break off from the needle, and they were analysed through Pendent_Drop plugin by ImageJ (imagej.net/plugins/pendent-drop).

In the experiment to investigate local fibre contact status with target surfaces, a glass capillary with outer diameter of 675 μm was placed on the surface of a microscopic glass slide (length 76 mm), and then a fibre was deposited onto the glass slide and the glass capillary. The fibre spinning and wetting process was in situ recorded. The glass slide was either treated to be hydrophilic (water contact angle roughly 0°) or hydrophobic (water contact angle roughly 90°), through 30 min of ultraviolet (UV) plasma treating or 20 s of Sigmacote soaking, respectively.

### Biocompatibility tests with chicken embryos

Wild-type fertilized chicken eggs, acquired from MedEgg Inc. Eggs, were stored in a monitored 15 °C fridge and incubated at 37.5 °C at around 45% humidity in egg incubators (Brinsea). The embryo extraction and ex ovo culture were performed as previously described^53^. PEDOT:PSS-based bioelectronic fibres were used for the chicken embryo experiments.

### Fabrication of fibre biopotential monitoring electrodes

For contact impedance and ECG measurements, a strip of copper tape with a wire to serve as connections to the outer circuit was first placed onto the nail of the index fingers of both hands of the volunteer. The PEDOT:PSS-based bioelectric fibres were then deposited onto both index fingers (left-hand index finger first and then right hand) of the volunteer for the measurements of contact impedance and ECG signals. An array of parallel bioelectronic fibres (an array of 180 parallel fibres across 9 mm, $\frac{N}{d}=\frac{180}{9\,{\mathrm{mm}}}$) were used. To create such an electrode, during fibre spinning the translational stage was set to move at a constant speed to deposit the fibre array to loop around the fingertip and the copper electrode on the nail. The interfacial impedance between the fibre and the skin decreases as the number of fibres *N* increases approximately linearly with time (see discussion notes in Supplementary Fig. 14). For this demonstration, the translation stage speed was set at roughly 50 μm s^−1^, thus a total distance of around 9 mm was covered over a roughly 3 min deposition time. Fibres on the left-hand index finger were used as the working electrode, and fibres on the right-hand index finger were used as the counter electrode. For EMG measurements, a strip of Kapton tape was first placed above the metacarpal bone of the thumb for insulation purposes and then a strip of copper tape with wire was then placed on top of the Kapton tape. Fibres were then deposited onto thumb-thenar muscle region. An array of parallel bioelectronic fibres (an array of 360 parallel fibres across around 2 cm, $\frac{N}{d} \approx \frac{360}{2\,{\mathrm{cm}}}$) were used, and, during deposition, the fibre spinning platform moved linearly at a constant speed of 50 μm s^−1^ to allow fibre deposition covering the muscle area. The loading of various weights was applied to the thumb.

The impedance was measured by a PalmSens4 Potentiostat, and the ECG and EMG signals were measured with Intan RHS Stim/Recording System. To compare the ECG signals obtained from the fibre and gel electrodes, both sets of ECG signals were simultaneously collected from the same individual. For working electrodes on the left hand, the fibre electrode was positioned on the fingertip of the index finger, and the gel electrode was placed on the fingertip of the middle finger. Additionally, a separate fibre electrode was placed to the fingertip of the right-hand index finger as a counter electrode. The signals exhibited high consistency with the correlation *P* values ranging from roughly 0.95 to 0.99 after applying the signal filtering. The ECG signals were filtered through a band-pass filter of 0.5–50 Hz and EMG signals were filtered through a band-pass filter of 0.5–500 Hz. The gel electrodes used as references were commercial disposable ECG Electrodes (ADInstruments). The Pearson correlation coefficient (*P*) between ECG signals was calculated by MATLAB.

### Fibre electrode stability and repairability study

The surface debonding and peeling experiment was performed by depositing an array of roughly 500 bioelectronic fibres onto a cherry tree leaf. Both ends of the bioelectronic fibres were attached and glued onto two parallel lifting arms, which lifted the fibre array to peel it off from the leaf at a constant speed of 50 μm s^−1^. The force was measured by a balance (Ohaus Scout Portable Balance, 120 g capacity, resolution 0.001 g) at one reading per second. Note that the ASTM D2861 test (90° peeling test) was not replicated here due to the difficulties of manipulating the thin substrate-free fibre arrays on a plant leaf.

In the mouse clicking experiment, the clicking force of the mouse (Onecall, catalogue no. CS33211) was measured by a force gauge (RS Component, FK 50, resolution 0.02 N). The mouse clicking speed was around one click per second. In the dry friction experiment, a cylindrical roller (outer diameter 2 cm) was 3D printed with polylactic acid or polylactide material and mounted on a servo motor (Parallax 6 V continuous servo, roughly 40 rpm). The speed of friction between the roller surface and the fingertip was roughly 4 cm s^−1^. The whole set-up was placed on top of a balance (Fisher Scientific, CSC 5000, resolution 1 g) to measure the normal pressing force. Dry friction experiments were performed in an air-conditioned room to minimize perspiration. The same experimental set-up was used in the wet friction experiments and deionized water was sprayed (roughly 0.2 ml) onto the fibre electrodes on the fingertip every 30 s (roughly every 120 cm friction distance). The cellulose-based protective fibre layer (an array of 90 parallel fibres across roughly 9 mm, $\frac{N}{d} \approx \frac{90}{9\,{\mathrm{mm}}}$) was deposited on top of the bioelectronic fibres at an angle of roughly 30°. Hand rinsing was performed with running tap water. In each rinsing cycle, the fingertip side was directly faced with running water for 10 s and then the nail side (with the fibre contact) was directly faced with running water for 10 s; this was followed by 10 s resting until the next rinsing cycle. The bioelectronic fibres were protected with a layer of cellulose-based fibres of an array of 90 parallel fibres across around 9 mm, $\frac{N}{d} \approx \frac{90}{9\,{\mathrm{mm}}}$, and then the fibre connection area on the nail was encapsulated by applying a cellulose-based liquid plaster film (nitrocellulose as the main solid ingredient, New-Skin Liquid Bandage Spray).

### Augmented touch

For the case of touch dual-ECG sensing, an array of bioelectronic fibres (180 parallel fibres across 3 mm, $\frac{N}{d}=\frac{180}{3\,{\mathrm{mm}}}$) were deposited onto the index fingers of person-i. During the measurements, bioelectronic fibres were tethered onto both the left- and right-hand index fingers of person-i to serve as working and counter electrodes, respectively. Then person-i touched person-ii’s index finger or wrist area so that the fibres were in direct contact with the skins of both people. Individual ECG signals of both people for validation were acquired as references using commercial disposable ECG gel electrodes at the same time (ADInstruments). The raw ECG signals were measured by the gel electrodes attached to individual people. The purpose of the individual ECG signal measured by gel electrodes (Supplementary Fig. 18a) is to validate that the signals measured from the fibres reflected the ECG of both people. The reconstructed dual-ECG signal in Fig. 4a was obtained by superimposing the individual ECG signals measured by the gel electrodes (with a scaling factor of 0.5).

### Dual-model sensing

An array of bioelectronic fibres (roughly 100 fibres parallel fibres across 0.5 cm) and an array of PEO-base pH-responsive fibres (~400 fibres parallel fibres across 0.5 cm) were looped onto the tip of the index finger separately. Two strips of copper tapes were placed on the nail to serve as electrical connections to measure the resistance change of the bioelectronic fibres. The resistance changes of the bioelectronic fibres on applying mists were acquired by a multimeter (Keysight, catalogue no. 34465A). An ultrasonic humidifier (VicTsing Essential Oil Diffuser) was used to generate humid mists of various pH levels. An acidic mist was generated from a solution by adding 100 μl of white vinegar into to every 10 ml of water (pH of the solution roughly 3); an alkaline mist was generated from a solution by adding 0.5 g of laundry powder (non-biological laundry powder, Sainsbury’s) into every 10 ml of water (pH of the solution around 12).

### Skin-gated OECT

The OECT measurement was commenced using a standard Source Measurement Unit (SMU) (catalogue no. 2612B, Keithley). Fibre arrays were deposited on the tip of a little finger. One SMU channel was connected through the fibre array channel to its counter electrode, and the other SMU channel was connected to a gate electrode through a single end connection (schematically shown in Supplementary Fig. 6). Both SMU channels were configured to share the common ground and have a constant current limit at 1 mA. The channel voltage was set at 1 V, and the gate voltage was pulsed between 0 to 0.5 V.

### UV-vis characterizations

The UV-visible light (vis) spectra were acquired with a JENWAY 7250 UV/Vis Spectrophotometer by depositing fibre arrays of various densities on glass cover slips.

### Plant interfaces

In the experiment of environmental ammonia sensing for plants, an orange LED (ROHM Semiconductor, catalogue no. SML-811DTT86A) was placed onto the leaf of an orchid plant with tweezers (without any adhesives), which was followed by orbital spinning of bioelectronic fibres that selectively ‘wrapped’ around the electrode contacts of the LED light along with the parts of the leaf surface (roughly 75 fibres in each array, and the length of fibres roughly 4 cm in each array). The fibre tethering provided both mechanical mounting support and electrical connections for the LED. In this case, the fibre arrays were deposited in a fanning pattern (fanning angle of roughly 15°, $\frac{N}{\theta } \approx \frac{75}{15^\circ }$ for each electrode contact) with the centre on the electrodes of the LED. The system was powered by a d.c. voltage supply (Digital Bench Power Supply 180 W, RS Component), and the driving voltage (~5–13 V depending on fibre array resistance: the electrical field did not exceed 6 V cm^−1^ in the fibre array) was tuned to just light up (turn on) the LED light before ammonia exposure (see detailed discussions on the operation of the warning system in Supplementary Fig. 22). Ammonia solution (2.3%) was sprayed onto the leaf of the plant to simulate the environmental ammonia contamination. The resistance change of the fibre array as a result of ammonia contact resulted in a change in LED brightness (which was recorded through a video). The bioelectronic fibres’ resistances in response to varied concentrations of ammonia vapour were tested an array of fibres with $\frac{N}{d} \approx \frac{75}{3\,{\mathrm{mm}}}$ and 20 mm in length on a microscope glass slide. The fibres, on the bottom surface of the glass slide, were placed above the ammonia solutions of various concentrations (1.7, 2.3, 2.8% in water) at a distance of 2 cm for 2 min.

### Reconfigurable fibre sensing interface

The steps of creating a reconfigurable fibre sensing interface on an orchid leaf is shown in Supplementary Fig. 24b. First, two orange LEDs (ROHM Semiconductor, catalogue no. SML-811DTT86A) were placed on the surface of an orchid leaf, followed by fibre tethering. Four arrays of bioelectronic fibres (roughly 60 fibres in each array with $\frac{N}{d} \approx \frac{60}{1\,{\mathrm{mm}}}$) were deposited to connect the electrodes of the LEDs, respectively (steps 1 and 2). Under this configuration, only one LED could be powered under one power supply channel. In the reconfiguration process (steps 3 and 4), a cotton bud with 70% ethanol was used to selectively erase unwanted fibre array traces, followed by tethering new fibre arrays to form a new sensing interface (the number of fibres *N* might need to be adjusted depending on the reconfigured fibre path, see additional discussion in Supplementary Figs. 22 and 24). Under the reconfigured circuit, both LEDs could be powered without need to change the outer circuit connections. A power supply was used (Digital Bench Power Supply 180 W, RS Component) to provide d.c. voltage (<13 V) in this experiment.

### Fibre tethering with e-textile

Stainless-steel yarns (Rapid Electronics, catlogue no. 87-6102, Light Stitches Conductive Thread Kit) were sewed to cover the fingertip region of white cotton gloves, followed by bioelectronic fibre tethering on top of the stainless-steel yarn region (180 fibres across around 5 mm distance, $\frac{N}{d} \approx \frac{180}{5\,{\mathrm{mm}}}$). During the biopotential measurement, the person wearing the glove put the finger onto the wrist area of the other person, with the fibre coated region directly in contact with the left-hand wrist of the other person. A hydrogel pad was connected to right-hand wrist of the other person to serve as the counter electrode. The stainless-steel yarns were connected to the working electrode of an electrophysiology machine (Intan RHS Stim/Recording System) for ECG measurement, and to a Potentiostat (PalmSens4) for contact impedance measurement. After measurement, the bioelectronic fibres were removed from the glove by brushing and recycled.

### Recycle of wasted bioelectronic fibres

The bioelectronic fibres were removed from the target objects (that is, leaf and e-textile glove) after use, and were then recycled for making conducting 3D printing inks. Once dry, the recycled fibres were redispersed into 70% ethanol (which also acted as a sterilizing medium) at a concentration of 1.5% w/w, and 1% of 400,000 Da PEO powders were also added. Then the dispersion was grinded for 5 min to form a homogeneous solution paste, which could be loaded into a syringe for printing.

### Ethics statement

Human participant experiments were performed with the approval of the Ethics Committee of the Department of Engineering at the University of Cambridge (7 July 2021, CUEDREC) and after obtaining informed consent from volunteers. No animal protocol was required for the chicken embryonic stages studied (less than 2 weeks) under the UK Animals (Scientific Procedures) Act 1986.

### Reporting summary

Further information on research design is available in the Nature Portfolio Reporting Summary linked to this article.

### Supplementary information

Supplementary Information Supplementary Figs. 1–25, Notes 1–8 and Tables 1 and 2.

Reporting Summary

Supplementary Video 1 Organic bioelectronic fibre tethering on different fingers.

Supplementary Video 2 Imperceptible fibre tethering.

Supplementary Video 3 A chicken’s embryo heartbeats with a fibre network.

Supplementary Video 4 Fibre tethering and contact dynamics.

Supplementary Video 5 Comparing fibre and commercial electrodes for fingertip-based ECG acquisition.

Supplementary Video 6 ECG monitoring with bioelectronic fibres under dry friction, humid, water-soaking and mild heat conditions.

Supplementary Video 7 Augmented mist sensing on a dandelion.

### Source data

Source Data Fig. 2 Raw data for Fig. 2a. Source Data Fig. 3 Raw data for Fig. 3b–h. Source Data Fig. 4 Raw data for Fig. 4. Source Data Fig. 5 Raw data for Fig. 5.

## Data Availability

Source data are provided with this paper. Source data are also available via Zenodo at 10.5281/zenodo.10808385 (ref. ^54^). Other data that support the findings of this study are available from the corresponding author upon reasonable request.
