# Supplementary material for: Imperceptible augmentation of living systems with organic bioelectronic fibres
Source: Nat Electron. 2024 May 24;7(7):586–97. doi: 10.1038/s41928-024-01174-4 (PMC11286532; doi:10.1038/s41928-024-01174-4)
Supplement: Supplementary file 1 — Supplementary Figs. 1–25, Notes 1–8 and Tables 1 and 2. [file 41928_2024_1174_MOESM1_ESM.pdf]

nature electronics

Supplementary information https://doi.org/10.1038/s41928-024-01174-4

Imperceptible augmentation of living systems with organic bioelectronic fibres

In the format provided by the authors and unedited

Table of contents

Supplementary Video 1: Organic bioelectronic fibre tethering on different fingers

Supplementary Video 2: Imperceptible fibre tethering

Supplementary Video 3: A chicken embryo's heartbeats with a fibre network

Supplementary Video 4: Fibre tethering and contact dynamics

Supplementary Video 5: Comparing fibre and commercial electrodes for fingertip-based ECG acquisition

Supplementary Video 6: ECG monitoring with bioelectronic fibres under dry friction, humid, water-soaking, and mild heat conditions

Supplementary Video 7: Augmented mist sensing on a dandelion

Supplementary Table 1: Comparing the imperceptible fibre augmentation with state-of-the-art on skin sensors and fibre printing techniques

Supplementary Table 2: Formats of bioelectronic fibre arrays designed for various applications

Supplementary Note 1: Estimation of power and materials consumption to create imperceptible augmentation devices via organic bioelectronic fibre tethering

Supplementary Note 2: Estimating the fibre deposition force

Supplementary Note 3: The drawing and drying process of a solution fibre investigated under an extension apparatus

Supplementary Note 4: Experimental characterisation of tethered fibre contact states and theoretical analysis comparing the driving capillary force for spreading of a wet fibre versus the resisting elastic force

Supplementary Note 5: Evaluating the fibre array tensile properties under different relative humidity environments

Supplementary Note 6: Breathable skin-gated OECT

Supplementary Note 7: Comparison of the environmental footprints between orbital spinning and microfabrication

Supplementary Note 8: Comparison of the environmental footprint between the fibre arrays in this work and the washing cycles of fabrics

**Supplementary Fig. 1:**	Single fibre deposition force measurement
**Supplementary Fig. 2:**	Bioelectronic fibre drying process
**Supplementary Fig. 3:**	Fibre surface tethering and contact
**Supplementary Fig. 4:**	Schematic illustration of the fibre wetting analysis model
**Supplementary Fig. 5**	Mechanical testing of the tensile properties of the fibre arrays under different relative humidity (RH) environments
**Supplementary Fig. 6**	Bioelectronic fibres as skin-gated OECT
**Supplementary Fig. 7:**	Rheological and surface tension characterisations of the fibre solution with reference composite solutions to identify the 'spinnable' solution property region for the orbital fibre spinning technique
**Supplementary Fig. 8:**	Fibre initiation and spinning process
**Supplementary Fig. 9:**	Fibre-to-circuit electrical connections
**Supplementary Fig. 10:**	Fibre array transmittance with various fibre spacings ($\frac{d}{N}$)
**Supplementary Fig. 11:**	Bioelectronic fibres interfaced with chicken embryos, and biocompatibility test
**Supplementary Fig. 12:**	Images showing the fibre tethering contacts on orchid flower petals
**Supplementary Fig. 13:**	SEM images and histograms showing fibre feature width (*w*) of bioelectronic and pH-responsive fibres in (a) surface-adhesion, and (b) suspension states
**Supplementary Fig. 14:**	Bioelectronic fibres on a fingertip for biopotential monitoring
**Supplementary Fig. 15:**	Comparing simultaneous ECG measurements from a fibre or a gel electrode for five volunteers (*P* represents data correlations*)
**Supplementary Fig. 16:**	Variations of the muscle EMG amplitude versus the weight of loading on hand
**Supplementary Fig. 17:**	The stability of bioelectronic fibres for biopotential monitoring during dry frictions, with simulated grease, or under heat, and water-soaking conditions

Supplementary Fig. 18: Surface adhesion characterisations and failure mechanisms of the bioelectronic fibre arrays on typical living structure surfaces under dry conditions

Supplementary Fig. 19: The effect of environmental disturbance on the stability of bioelectronic fibres and strategies to mitigate the effect

Supplementary Fig. 20: Scratching and water rinsing tests for fibre electrodes with encapsulated fibre-to-circuit connections

Supplementary Fig. 21: Identifying individual ECG signals from dual-ECG sensing

Supplementary Fig. 22: Bioelectronic fibres as plant interfacing sensor arrays

Supplementary Fig. 23: Cyclic voltammetry measurements of bioelectronic fibre arrays

Supplementary Fig. 24: Reconfiguring bioelectronic fibre sensing interface

Supplementary Fig. 25: Bioelectronic fibre network stretchability characterisations

References

Supplementary Video 1: **Organic bioelectronic fibre tethering on different fingers**

Bioelectronic fibre tethering process on different fingers with a laser pointer being used to indicate the fibre deposition positions. The laser pointer is aligned with the plane of the needle and the rotating arm; thus, the laser spot would indicate the real time fibre deposition location. This could be helpful especially when repairing and upgrading the fibre structures on-demand, because the fibres are small and sometimes difficult to see with naked eyes.

Supplementary Video 2: Imperceptible fibre tethering

The compound leaves of *Mimosa pudica* plant would fold inward and droop when touched or shaken[1]; thus, it is chosen as an exemplary target object to demonstrate that the fibre tethering process would induce minimal perturbance, and thus is imperceptible to the target objects. Bioelectronic fibres were first spun onto a leaf of the *Mimosa pudica* plant for around 90 seconds, the leaf did not fold or change in shape during the whole fibre deposition process. Afterwards, the same leaf was touched by fingers gently. Upon hand touching, the leaf folded immediately.

Supplementary Video 3: **A chicken embryo's heartbeats with a fibre network**

The day-3 chicken embryo was directly extracted from an egg, and the whole yolk with the embryo was placed inside a petri dish. Bioelectronic fibres were then spun onto the surface of the chicken embryo directly under room temperatures, and the process of fibre spinning lasted for around 5 minutes. Afterwards, the embryo was put back to a 37 °C incubator for 15 minutes before imaging the heartbeat. (Typical results from N>5 independent experiments on different embryos)

Supplementary Video 4: Fibre tethering and contact dynamics

This video shows the real-time fibre wetting process upon fibre tethering, where a bioelectronic fibre is spun onto a substrate made of a glass microscope slide with a round glass capillary placed on top.

Supplementary Video 5: Comparing fibre and commercial electrodes for fingertip-based ECG acquisition

This video shows the experimental configurations for acquiring ECG signals in real-time: Fibre and gel electrodes were placed onto the index and middle fingers respectively on the left hand as working electrodes, and a separate fibre electrode on the right hand index finger serve as a counter electrode.

Supplementary Video 6: ECG monitoring with bioelectronic fibres under dry friction, humid, water-soaking, and mild heat conditions

Clear ECG signals were measured from the bioelectronic fibres on a fingertip during dry friction wear, water soaking, ~ 90 % relative humidity, or mild heat at ~ 40 °C environments. For dry friction, a normal force of ~ 2 N was applied, and the surface of the plastic roller was

moving at a speed of 4 cm/s. A reference friction experiment was presented where the simultaneous ECG measurements from the fibre electrode under friction were compared to unperturbed fibre and gel electrodes. For the mild heat environment, the fingertip with bioelectronic fibres was placed ~ 30 cm in front of a 100-watt infrared lamp. No apparent distortion to the fibre patterns or signal deterioration was observed during the above wearing processes.

Supplementary Video 7: Augmented mist sensing on a dandelion

An array of bioelectronic fibres could be tethered onto a dandelion to monitor environmental mist interactions. During the fibre spinning, a bug living on the dandelion is seemingly not disturbed. Both ends of this bioelectronic fibre array are connected to a multimeter, which monitors the resistance change of the fibres. When a water mist flows over the dandelion, it could trigger the resistance increase of the bioelectronic fibres.

Supplementary Table 1 | Comparing the imperceptible fibre augmentation with state-of-the-art on skin sensors and fibre printing techniques. The test condition is highlighted when the durability tests were performed in situ on the living skins of the target objects, because such test conditions best characterise the stability of the devices during actual application scenarios. Upon device attachment/deployment onto living skins, the living objects' posture and movement could influence the outcome of the device interface. During the wearing, living skins would naturally produce sebum and undergo trans-epidermal water loss, which could also influence the device performance. The device life cycle sustainability refers to the environmental impact of a device throughout its lifetime; it is evaluated by considering the criteria of 'Eco-material' (i.e. use of earth-abundant, biologically-derived or degradable raw materials), 'Reduce' (i.e. minimising total embodied material, energy usage and emissions during fabrication), 'Repair' (readily repairable or reconfiguration for other purposes), and 'Recycle' (of extracted materials, or re-use material constituents or components at the end of the device service life). The *supporting data* leading to the electromechanical performance summary are highlighted in grey.

**Reference**	**Material and interface format**		**Fabrication for scalable customisation**	**Bio-imperceptibility ****	**Environmental electromechanical performance (*e.g.*, stability)**			**Target applications**
	**Sensing interface**<br>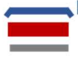 	**Electrical connection**<br>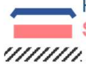 			**Unperturbed**	**Surface contact wear**<br>**Dry** **Humid** **Soaked**	**Stretch/ compress (Dry condition)**	**Device life cycle sustainability**
**This work**	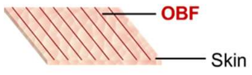<br><br>(OBF: Organic bioelectronic fibre)<br>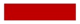<br>Fully-exposed OBF & contact	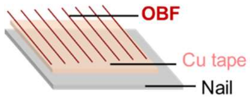<br><br>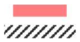	**High**<br>- In-situ tethering, physically adapting to target surface<br>- One-step circuitry coupling<br>- Over 90% success in device deployment*	**High**<br>- Fibre thickness and width ~ submicron to ~ microns<br>- Tethering force ~ 10 μN<br>- Network opening 10s μm to ~1 mm<br>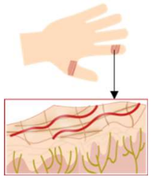	**Water soaked/ 90% humidity/ Heat ~ 40 °C** <u>(fingertip)</u><br>30 min, $\frac{Z}{Z_0}$ ~1<br>*Fig. 3f*<br>*SI-Fig. 17b*<br>**Simulated grease** (*e.g.*, Vaseline®, <u>fingertip)</u>, $\frac{Z}{Z_0}$ ~1<br>*SI-Fig. 17a*<br>**Ambient wear** <u>(fingertip)</u><br>> 6 hr, $\frac{SNR}{SNR_0}$ ~1<br>*Fig. 3f*	**Clicking** *Fig. 3f* <u>(fingertip/plastics)</u><br>F_N ~1.5 N<br>6,000 taps, $\frac{Z}{Z_0}$ ~1<br>**Friction** *Fig. 3f* <u>(fingertip/plastics)</u><br>F_N ~3 N, D=25m<br>$\frac{Z}{Z_0}$ ~1.3<br>**Scratching** *SI-Fig. 20a* <u>(nail/fabrics)</u><br>F_N~0.5 N, 1,000 cycles<br>$\frac{Z}{Z_0}$ ~1.4<br>**Office work** *SI-Fig. 19c* <u>(fingertip/keyboard)</u><br>1 hr, $\frac{SNR}{SNR_0}$ ~0.2<br>**Wet friction** *Fig. 3g, SI-Fig. 19a-b* <u>(fingertip/wet plastics)</u><br>F_N ~ 0.5 N, D =8m, $\frac{Z}{Z_0}$ ~22	**Stretching parallel fibres** (on Ecoflex)<br>ε =5%, 500 cycles<br>$\frac{R}{R_0}$ ~1.3<br>**Stretching orthogonal fibre network** (on Ecoflex)<br>ε =15%, 500 cycles<br>$\frac{R}{R_0}$ ~1.1<br>*SI-Fig. 25*	- Imperceptible and breathable biopotential (ECG, EMG) sensing, and skin-gated OECT on fingertips<br>- Augmented fingertip touch for dual-ECG sensing<br>- Augmented fingertip perception for environmental wetness<br>**Eco-material**<br>**Reduce**<br>**Repair**

	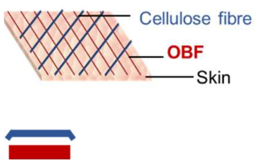 Fibre protected OBF & contact	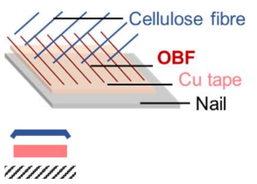			/	**Office work** *SI-Fig. 19d* (fingertip/keyboard)<br>1 hr, $\frac{SNR}{SNR_0}$ ~0.9<br>**Wet friction** *Fig. 3g, SI-Fig. 19a-b* (fingertip/wet plastics)<br>F_N ~ 0.5 N<br>D =8m, $\frac{Z}{Z_0}$ ~1.3	/	- Imperceptible and breathable biopotential monitoring from fingertips<br>**Eco-material**<br>**Reduce**
	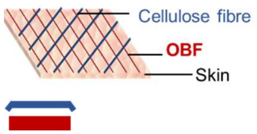 Fibre protected OBF & film protected contact	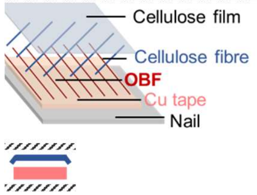			/	**Scratching** *SI-Fig. 20a* (nail/fabrics)<br>F_N~3 N, 1,000 cycles<br>$\frac{Z}{Z_0}$ ~1<br>**Rinsing** *Fig. 3h* (fingertip/running water)<br>30 s rinse, 10 cycles<br>$\frac{Z}{Z_0}$ ~1.1	/	- Daily disposable, imperceptible biopotential monitoring<br>**Eco-material**<br>**Reduce**
	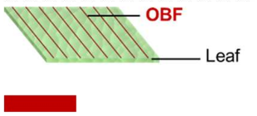	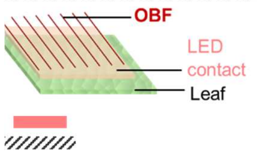			**80 % humidity** *SI-Fig. 22* (on glass slides)<br>1 hr, $\frac{R}{R_0}$ ~1	**Water Spray** *SI-Fig. 22* (leaf/water droplets)<br>$\frac{R}{R_0}$ ~1	/	- Large-area, photosynthesis compatible environmental sensors on plants<br>- Reconfigurable sensor network coupling<br>**Eco-material**<br>**Reduce** **Repair**<br>**Recycle**
**A.** 2	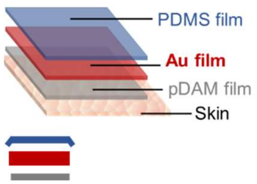	Connected to two Cu wires via liquid metals 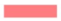	**Low**<br>- Micro-fabrication<br>- Multi-step circuitry coupling	**Limited**<br>- Film thickness ~1.3 µm<br>- No open porosity	**In Saline** (on a substrate)<br>937 hr, $\frac{Z}{Z_0}$ ~1<br>**Ambient wear** (wrist), 8 hr<br>$\frac{SNR}{SNR_0}$ ~1	**Rinsing/swimming** (wrist/running water)<br>cycle not reported<br>$\frac{SNR}{SNR_0}$ ~1	**Stretching** (standalone film)<br>ε =100%, 5,000 cycles, $\frac{R}{R_0}$ ~1	- ECG and motion sensor on wrist<br>- Implantable nerve electrodes<br>. / .

Reference	Material and interface format		Fabrication for scalable customisation	Bio-imperceptibility **	Environmental electromechanical performance (*e.g.*, stability)			Target applications
	Sensing interface 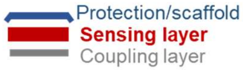	Electrical connection 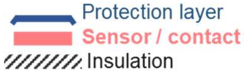			Unperturbed	Surface contact wear<br>Dry — Humid — Soaked	Stretch/ compress (dry condition)	Device life cycle sustainability
**B.** 3	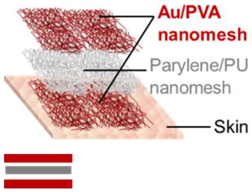	Via Cu wires using adhesive surgical tapes	**Low**<br>- Micro-fabrication<br>- Multi-step transfer and assembly	**Partial**<br>- Mesh thickness ~ 10s µm<br>- Random fibre section overlay<br>- Porosity submicron to ~microns 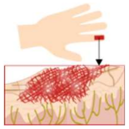	/	**Pressing**<br>(on a pressing rig)<br>P = 19.6 kPa<br>1,000 times<br>$\frac{\Delta C}{C_0}$ ~ stable<br>**Rubbing**<br>(on PU sheet/PU ball)<br>F_N = 5 N, 300 times<br>$\frac{C}{C_0}$ ~1, $\frac{\Delta C}{C_0}$ drops 9.7 %	**Stretching**<br>(nanomesh on a elastomer)<br>ε =14%<br>1 cycle<br>$\frac{C}{C_0}$ ~0.9	- On skin pressure sensor for fingertip in dry and non-sweating conditions<br>. / .
**C.** 4	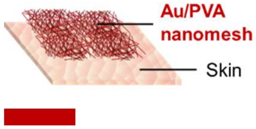	Connected to a flexible silver adhesive, which is connected to a e-textile glove via conductive threads	**Low**<br>- Micro-fabrication<br>- Delicate transfer and assembly needed	**Partial**<br>- Nano-mesh thickness-submicron<br>- Random fibre section overlay<br>- Porosity submicron to ~ microns 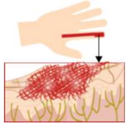	/	**Rubbing**<br>(finger/plastic)<br>150 kPa, 20 times<br>No physical damage (electrical performance not reported)	**Finger clenching**<br>(finger)<br>10,000 cycles:<br>$\frac{R}{R_0}$ ~2.7<br>**Stretching**<br>(on a 15 % pre-stretch PU sheet)<br>ε =25% 500 cycle<br>$\frac{G}{G_0}$ drops 8 %	- Hand movement, temperature, and pressure sensor<br>- Wearable biopotential sensor<br>. / .

**D.** 5	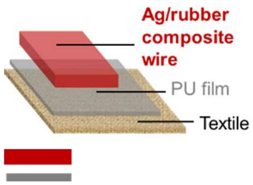	Via Cu tapes 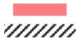	**Medium**<br>- ex situ 3D printing<br>- Requiring post-processing	**Limited**<br>- Sensors printed on fabrics	/	/	**Stretching**<br>(standalone wire)<br>ε =50%<br>100 cycles<br>$\frac{R}{R_0}$ ~100	- Wearable pressure and temperature sensor<br>- Robotic interface<br>. / .
**E.** 6	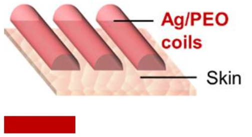	Directly connected to the Ag/PEO coils 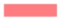	**Medium**<br>- In situ and adaptive 3D printing<br>- Demanding target tracking	**Limited**<br>- Coil width hundreds µm – millimetres<br>- Network opening ~ millimetres<br>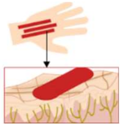	**100% humidity** <u>(back of hand)</u><br>20 min for 4 cycles,<br>$\frac{Z}{Z_0}$ ~1	/	**Bending**<br>(on artificial skins)<br>r=52 mm, 1,000 cycles, good adhesion<br>($\frac{Z}{Z_0}$ not reported)	- 3D printing wireless powered circuits on hand<br>**Recycle**
**F.** 7	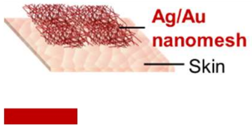	Via a nanomesh connector	**Medium**<br>- In situ spraying via masking for pattern creation	**Partial**<br>- Nano-mesh thickness ~ microns<br>- Random fibre section overlay<br>- Porosity submicron to ~ microns<br>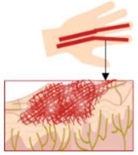	**Heat ~ 40 °C / In water / 3 hr ambient wear** <u>(back of hand)</u><br>Hand motion recognition unaffected<br>($\frac{R}{R_0}$ not reported)		**Stretching**<br>ε =30%<br>5,000 cycle<br>$\frac{R}{R_0}$ ~1	- Strain sensor for hand motion monitoring<br>. / .
	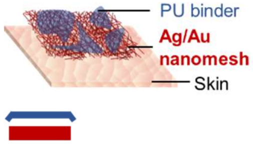	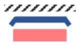			/	**Rubbing**<br>(porcine skin/rubber)<br>F_N =0.2 N<br>1,500 times, $\frac{R}{R_0}$ ~1.2<br>**Rinsing**<br><u>(back of hand /running water)</u><br>20 s rinse, 4 cycles<br>$\frac{R}{R_0}$ ~1.1	/	- Daily activity hand motion monitoring<br>. / .

Reference	Material and interface format		Fabrication for scalable customisation	Bio-imperceptibility **	Environmental electromechanical performance (e.g., stability)			Target applications
	**Sensing interface**<br>Protection/scaffold<br>Sensing layer<br>Coupling layer	**Electrical connection**<br>Protection layer<br>Sensor / contact<br>Insulation			**Unperturbed**	**Surface contact wear**<br>Dry – Humid – Soaked	**Stretch/ compress (dry condition)**	**Device life cycle sustainability**
**G.** 8	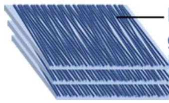	/	**Low**<br>**-** ex situ Rotary jet spinning (collection mandrel needed)	**Limited**<br>- dense fibrous mesh of 10 s µm<br>- Porosity ~ microns	/	/	/	- Fibrous scaffolds for heart models<br>/
**H.** 9	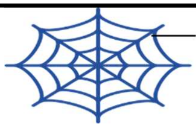	/	**Medium**<br>- ex-situ 3D printing	**Limited**<br>- Fibre width hundreds µm<br>- Network opening 10s mm	/	/	/	- Structural mimic of spider webs<br>/
**I.** 10	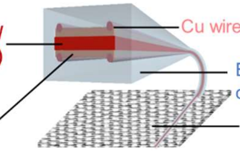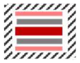		**Low**<br>**-** Thermal drawn 'thermally-drawn e-fibres' (fibre structure depends on the preform)	**Limited**<br>**-** Fibre width ~ millimetres	/	**Machine wash**<br>(fibre embroidery in fabrics /running water)<br>10 cycles,<br>$\frac{C}{C_0}\sim 1$	**Bending**<br>(fibre on a film)<br>min. r =1.2 cm<br>1,000 cycles<br>$\frac{C}{C_0}\sim 1$<br>**Twisting**<br>(standalone fibre)<br>Max. angle 540°<br>1,000 cycles<br>$\frac{C}{C_0}\sim 1$	- e-Fabrics with sensitive audible microphone function<br>/

* **Device deployment success rate** is defined as the rate of effective fibre formation per spinning rotation

**** Criteria for bio-imperceptibility**

<u>- High</u> (network openness > 50 µm; thickness < 10 µm)

This criterion is set by considering human sensory system and functions of skin. In brief, a network/mesh is considered as fully skin imperceptible if it simultaneously fulfils the conditions of: (1) network/mesh opening between fibres (openness) greater than ~ 50 µm (c.f., the sweat gland pore size), but smaller than 1 mm (c.f., the fingertip receptor field); (2) width of individual fibres and thickness of the network/mesh smaller than ~ 10 µm (such that individual skin cells are mostly exposed through the open network, and the fingerprint ridge features are not compromised).

<u>- Partial</u> (porosity < microns and thickness < 10 µm)

<u>- Limited</u> (porosity < ~1nm or thickness > 10s µm)

Order of references in the above table:

A. Jiang, Zhi, et al. Nature Electronics 5.11 (2022): 784-793. [2]
B. Lee, Sunghoon, et al. Science 370.6519 (2020): 966-970. [3]
C. Miyamoto, Akihito, et al. Nature nanotechnology 12.9 (2017): 907-913. [4]
D. Matsuhisa, Naoji, et al. Nature materials 16.8 (2017): 834-840. [5]
E. Zhu, Zhijie, et al. Advanced Materials 30.23 (2018): 1707495. [6]
F. Kim, Kyun Kyu, et al. Nature Electronics 6.1 (2023): 64-75. [7]
G. Chang, Huibin, et al. Science 377.6602 (2022): 180-185. [8]
H. Qin, Zhao, et al. Nature communications 6.1 (2015): 7038. [9]
I. Yan, Wei, et al. Nature 603.7902 (2022): 616-623. [10]

List of abbreviations:

C – capacitance
F_N – normal force
G – conductivity
OBF – organic bioelectronic fibre
R – resistance
PCL – Polycaprolactone
PDMS – Polydimethylsiloxane
PE – Polyethylene
PLA – Polylactic acid
PU – Polyurethane
PVA – Poly(vinyl alcohol)
P(VDF-TrFE) – poly(vinylidene fluoride-co-trifluoroethylene)
SNR – signal to noise ratio
r – radius
Z – impedance
ε – normal strain

Supplementary Table 2 | Formats of bioelectronic fibre arrays designed for various applications. A summary of the bioelectronic fibre number density ($\frac{N}{d}$), fibre network opening (average fibre spacing, $\frac{d}{N}$), and estimated transparency in various bioelectronic applications demonstrated in this work, in comparison to the size of biological features on human skins.

**Objects**	**Application scenarios**	**Figure**	$\boldsymbol{\frac{N}{d}}$	$\boldsymbol{\frac{d}{N}}$ **Mesh opening/ average fibre spacing (µm)**	**Reason for choosing the ($\frac{N}{d}$)***	**Estimated transparency (%)****
Bioelectronic fibres	ECG measurement from fingertip	Fig. 3c	$\sim\frac{180}{9\ mm}$	~ 50	From Fig. 3b, the contact impedance decreases to ~ 10^4 Ω with ~ 180 fibres, and spinning more fibres would not significantly improve the contact impedance further.	91 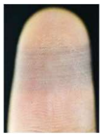
	EMG measurement from thumb muscle	Fig. 3d	$\sim\frac{360}{2\ cm}$	~ 60	Large fibre number and deposition width were chosen to ensure good fibre coverage on the thumb muscle region.	90 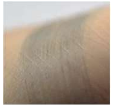
	Augmented touch on fingertip (dual-ECG sensing)	Fig. 4a	$\sim\frac{180}{3\ mm}$	~ 20	A narrow deposition width was used to ensure all fibres could be in contact with the other person's skin when touching.	88 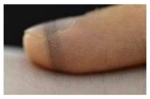
	Skin-gated OECT on fingertip	Fig. 4b	$\sim\frac{15}{3\ mm}$	~ 200	A small fibre number was used to enhance the response time (further analysis in Supplementary Note 6).	98 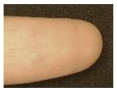
	Dual-model sensing on fingertip (moisture)	Fig. 4c	$\sim\frac{100}{5\ mm}$	~ 50	The resistance of ~ 100 fibres is ~ 10 kΩ, which is measurable by common multimeter.	90 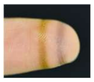
	Microelectronic integration on leaf	Fig. 5b, Fig. 5e	$\sim\frac{60-75}{1-3\ mm}$	~ 20-75	~ 60-75 fibres to allow a desirable driving voltage; a narrow fibre array width to ensure direct connection with a small LED.	88-92 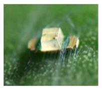
	Ammonia sensing on leaf	Fig. 5c	$\sim\frac{75}{15°}$	-	A fanning fibre array was used to enlarge the fibre array sensing area.	-
Size of biological features on human skins	sweat pore size			~ 60-80 µm		
	Fingerprint ridge spacing			~ millimetre range		
	Single skin cell size			~30 µm		
	Receptor fields on fingertip			~ millimetre range		

*in general, the number of fibres *N* is mainly determined by the required contact impedance or circuity level fibre array resistance, and the fibre array width *d* is customised according to the desired fibre array surface coverage and the choice of electrode contacts coupling to other devices or peripheral circuitry.

**this is estimated based on the relationship between the fibre number density and the transparency (Fig. 2a and Supplementary Fig. 10).

Supplementary Note 1: Estimation of power and materials consumption to create imperceptible augmentation devices via organic bioelectronic fibre tethering

For the estimation of the power consumption of the base unit of the fibre spinning set-up, the fibre production and the solution feeding mechanisms are considered. For the fibre production mechanism, the rotating arm operates at 4-6 V with 120 mA current according to the manufacturer, corresponding to 0.6 W; and an Arduino UNO board was used to control the rotating arm, and the board usually operates with no more than 0.4 W[11]. For the solution feeding mechanism, the micro air pump operates at 45-60 mA according to the manufacturer, corresponding to 0.54-0.72 W under 12 V voltage supply. Thus, one base unit of the fibre production and solution feeding mechanisms requires around 2 W power consumption. During the fibre spinning process, the flowrate of the solution feeding is adjusted at around 50-70 µL/hr (mean ~60 µL/hr), which corresponds to around 1 µL of solution per minute of fibre spinning. Considering typical bioelectronic fibre tethering for 2-5 minutes, corresponding to ~ 2-5 µL of solution being used. The solid matter concentration of all the solutions used is below 5 % (w/w); thus, the dry mass of the bioelectronic fibres is estimated to be ~ 0.1-0.3 mg per device. When corresponding to the fibres used for per device, the fibre number density commonly used are $\frac{N}{d} \sim \frac{100}{3mm}$ to $\frac{300}{20mm}$ for each device (NB. in most applications, the total fibre number used are ~100-300 fibres; and only in the OECT case, 15 fibres were used). It is to note that the solution usage and dry mass calculated here are the total embodied material usage (*i.e.*, including solutions wasted within the needle and the rotating arm).

Supplementary Note 2: Estimating the fibre deposition force

Cantilever experiment was set up to characterize the dynamic fibre printing process. The cantilever was made of an elastic quartz micropipette-filling capillary (inner diameter of 100 µm and outer diameter of 164 µm; World Precision Instruments). The elastic behaviour of the cantilever (force at the free end tip versus tip deflection) had been characterized previously[12]. The cantilever deflection versus force could be estimated by *Force(µN) = 9.46 × deflection (mm)* where the force is causing deflection. During the experiment to characterise fibre deposition force, a single fibre was spun onto the free end tip of the cantilever, and the deflection of the tip was recorded through video and then measured that could be correlated to force. The bioelectronic fibre caused ~ 1.2 mm deflection of the cantilever, corresponding to less than 12 µN of deposition force.

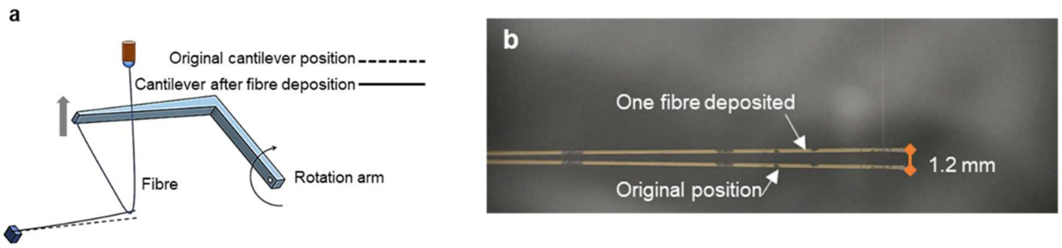

Supplementary Fig. 1 | Single fibre deposition force measurement. a, Sketch showing the experimental set-up where a single fibre is deposited onto the free end tip of a suspended elastic cantilever, causing the deflection of the cantilever. **b,** Overlayed images showing the cantilever deflection due to the deposition of a single bioelectronic fibre.

Supplementary Note 3: The drawing and drying process of a solution fibre investigated under an extension apparatus

As shown below an image sequence taken by a high-speed camera shows the bioelectronic fibre drying process upon solvent (water) evaporation. In the fibre spinning process, it takes about 0.5 second for each fibre to be stretched in air. With an initial fibre width of ~ 80-100 µm, it would take more than 5 seconds for the solvent to evaporate under normal room conditions.

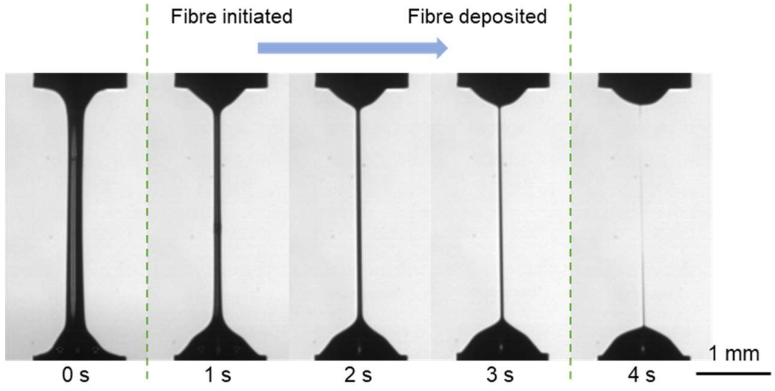

Supplementary Fig. 2 | Bioelectronic fibre drying process. Image sequence showing the drying process of a single bioelectronic fibre, from the moment of being initiated.

Supplementary Note 4: Experimental characterisation of tethered fibre contact states and theoretical analysis comparing the driving capillary force for spreading of a wet fibre versus the resisting elastic force.

i)- Characterisation of tethered fibre contact states

We investigate the state of fibre tethering where solution fibres create contacts with local surfaces in the case when the surface is solid and convex. Evaluating fibre diameter changes over time indicates that for the bioelectronic fibre formulation, abundant residual water remains in the 'wet fibre' upon surface tethering; thus, a dominant Wenzel-like[13] state is resulted. For a substrate feature of hundreds of microns in diameter (i.e., representing topographical features of finger ridges and orchid flower petals), differences in surface hydrophobicity only have minor effects on the relative contact line coverage likely due to the amphiphilic nature of PEO[14].

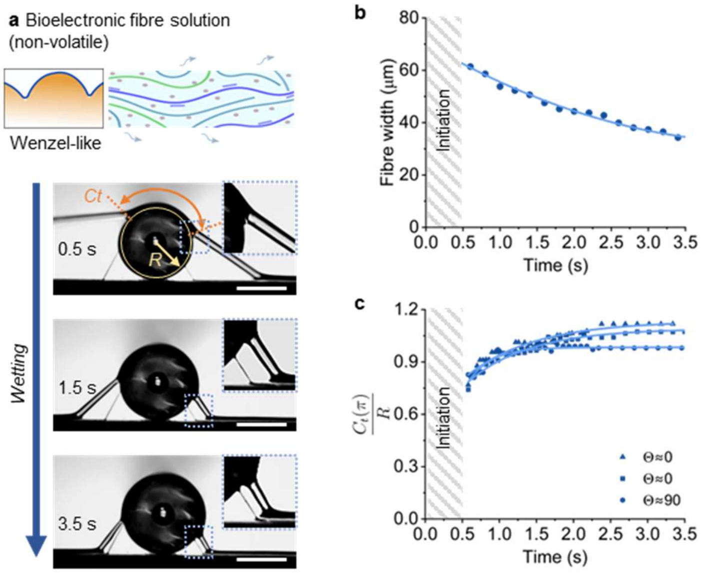

Supplementary Fig. 3 | Fibre surface tethering and contact. a, Images showing the time-dependent wetting process for a bioelectronic fibre (scale bars, 500 μm). The zoom-in images compare a bioelectronic fibre undergoing continuous wetting for ~ 3.5 s after in-air spinning, and a previously deposited dried fibre with much thinner diameter. **b,** Plots of fibre widths versus time upon fibre tethering on a glass surface for the two solutions. **c,** Plots of fibre solutions' normalised contact line length ($\frac{C_t(\pi)}{R}$) versus time for the two solutions; in particular for the bioelectronic fibre solution, treated glass surfaces with various hydrophilicities are tested. C_t is the time-dependent contact line length, and R is the radius of the curved glass surface, as defined in (**a**); and Θ in (**c**) indicates the water contact angle for the treated glass surface.

ii)- Theoretical analysis comparing the driving capillary force for spreading of a wet fibre versus the resisting elastic force

Theoretical analysis is used to compare the driving capillary force for the spreading of a wet fibre versus the resisting elastic force. To conform to sub-millimetric reliefs, the fibre must wet the substrate so it can spread into the grooves. The driving capillary force for spreading of the fibre of radius R may be given by $F_c \sim \gamma R$, where γ is the liquid surface tension (assuming complete wetting). Concomitantly to spreading within a groove, say a "V-shaped" cut of depth h on the surface as shown in the figure below, the strain of the fibre could be shown as for it to reach a depth d (if the angle of the V-shaped groove is about $\pi/3$):

$$\varepsilon = \frac{d}{h}\left[\frac{1}{\sin\left(\frac{\theta}{2}\right)} - 1\right]$$

So that the strain satisfies $\varepsilon \sim d/h$. The dominating force resisting this elongation is thus elastic, $F_e \sim GR^2\varepsilon$, where G is the fibre's elastic (or storage) modulus. Balancing both forces, the fibre will reach a relative depth $d/h \sim \gamma/(GR)$. If $\gamma \gg GR$, the fibre will completely conform to the jagged surface. On the other hand, the fibre will remain suspended if $\gamma \ll GR$.

This analysis shows that the driving capillary force would dominate for the spreading of a wet fibre. Hence, for the bioelectronic fibre formulation, both experimental and theoretical evidence indicate that intimate contacts over hundreds of microns of topographical features are expected to form on convex and solid structures.

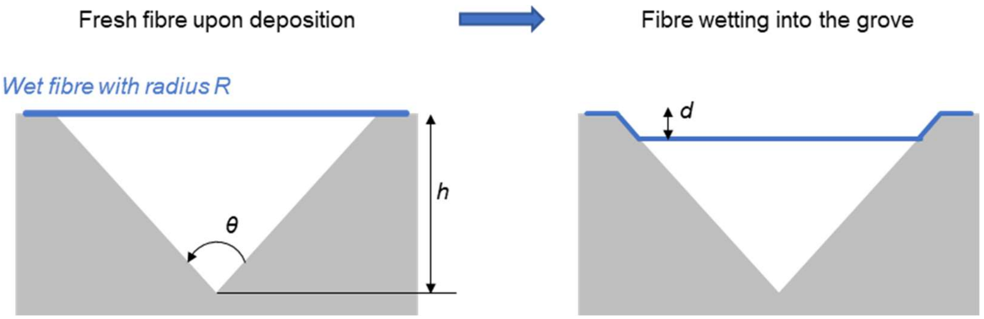

Supplementary Fig. 4 | Schematic illustration of the fibre wetting analysis model.

Supplementary Note 5: Evaluating the fibre array tensile properties different relative humidity environments

An array of parallel suspended fibres ($\frac{N}{d} \sim \frac{400}{4\ cm}$) was first deposited on a frame with a gap distance of 20 mm, and the frame was mounted onto a vertical translational stage (Thorlabs MTS50-Z8), moving at a constant speed of 50 µm/s upwards. The rising suspended fibre array was intercepted in the middle by a weight placed on a balance (Ohaus Scout Portable Balance, 120 g Capacity, 0.001 g Readability). The set-up is schematically shown in Supplementary Fig. 5a. The reading on the balance reflects the forced induced by the deformed fibre array. The strain of the fibre array could be calculated through:

$$Strain = \frac{2\sqrt{D^2 + \left(\frac{L}{2}\right)^2} - L}{L}$$

where D is the displacement of the vertical translational stage and L is the original length of the fibre array. The whole set-up is placed inside a humidity-controlled chamber for adjusting the relative humidity (RH).

The tensile strength of the bioelectronic fibres would weaken at high humidity levels (*i.e.,* RH ~ 70 %), and this enables fibres at defined regions to be easily erased off on demand upon wetting (as exemplified in Supplementary Fig. 5d). Such mechanical remodel-ability further offers the possibility for conveniently remodel the in situ circuit patterning. For example, the tethered bioelectronic fibre circuit could be selectively erased off to create connection openings.

In comparison, the cellulose-based fibres possess almost 10 times higher tensile strength than the bioelectronic fibres, and their mechanical properties are insignificantly affected by environmental humidities for the duration of the measurements (~ 10 minutes). Thus, the cellulose-based fibres could be used as a protective fibre layer on top of bioelectronic fibres as an approach to enhance the environmental stability of the system (see examples in Supplementary Fig. 19).

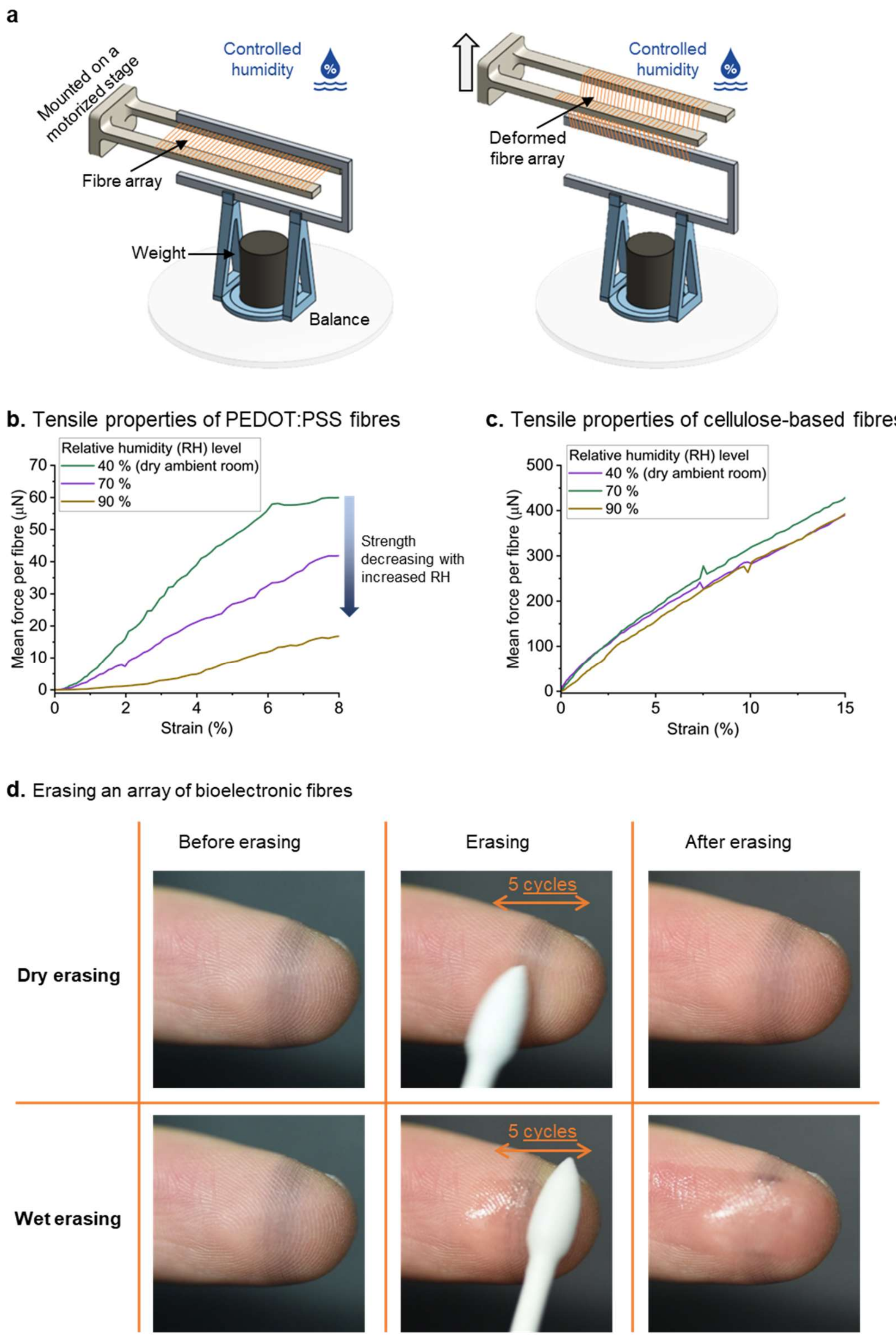

Supplementary Fig. 5 | Mechanical testing of the tensile properties of the fibre arrays under different relative humidity (RH) environments. a, Schematic drawings showing the set-up to measure the tensile properties of a fibre array produced by fibre tethering under a controlled humidity chamber. **b-c,** Average tensile force per fibre versus strain at different RH levels for (b) bioelectronic fibres and (c) cellulose-based fibres (the average ambient room humidity level in the lab is ~ 40 % RH, which is considered as 'dry' environments). **d,** Selectively erasing a section of bioelectronic fibre array on the fingertip with a cotton bud in

dry versus wet conditions. In the dry erasing test, the cotton bud was used as it is, and in the wet erasing test, the cotton bud was soaked with water for erasing. Each erasing was conducted by moving the cotton bud on the fingertip back and forth for 5 cycles, with a pressing force of ~ 1 N.

Supplementary Note 6: Breathable skin-gated OECT

The design of skin-gated OECT with an array of bioelectronic fibres is schematically shown in Supplementary Fig. 6a. The variable permeability of the human skin epidermis layer[15], in combination with the ionic solution properties of interstitial fluid, allow gating circuitry to form across human body surface for transistors that rely on interfacial charge exchange to operate[16]. In the context of OECT, the skin thus constitutes the "electrolyte" component for the OECT architecture[17]. Supplementary Fig. 6b shows the OECT responses versus the number of fibres in the channel with exponential fitting curves, and the time constants τ of the fitting curves are shown in Supplementary Fig. 6c (exponential fitting and time constant τ are generally used to characterise the OECT response behaviour[18]). It is seen that the OECT response would slow down with increasing number of fibres in the channel, especially when the fibre number exceeds 20. Thus, normally a fibre array of ($\frac{N}{d} = \frac{10}{3\,mm}$ to $\frac{20}{3\,mm}$) is used as the channel. With ~ 15 fibres in the channel, the time constant τ of the OECT response behaviour is ~ 12 s, and the response time could be estimated in the range of 60 s, similar to other patch-like OECTs of centimetre channel length[19]. Such a skin-gated low-frequency OECT could be useful for future clinical applications in measuring quasi-static continuous biological signals with long fluctuation time in hours, such as for hydration and circadian rhythm monitoring[20,21].

As for the operational safety considerations, the applied gate voltage does not exceed 0.5 V, and the gate and channel currents have been measured as shown in Supplementary Fig. 6d. With 0.5 V gate voltage, the maximum power through the channel does not exceed 20 μW, which would not cause overheating. The gate current that passes through the human body does not exceed 1 μA, which is 3 orders of magnitude lower than the safety current limit for human body (muscle let-go current)[22]. In addition, such voltage and current levels used in this work are much lower than commercial deep brain stimulation (2.5-3.5 V[23]) and transcutaneous electrical nerve stimulation (up to 30 V[24] and 80-100 mA[25]), and these devices usually work in long-term manners either implanted or directly interfaced with human skins. Finally, the channel current does not exceed 50 μA. The gate voltage, channel and gate currents of the skin-gated OECT in this work compare similar to or lower with those reported human-interfaced OECT devices from literature[16,26,27].

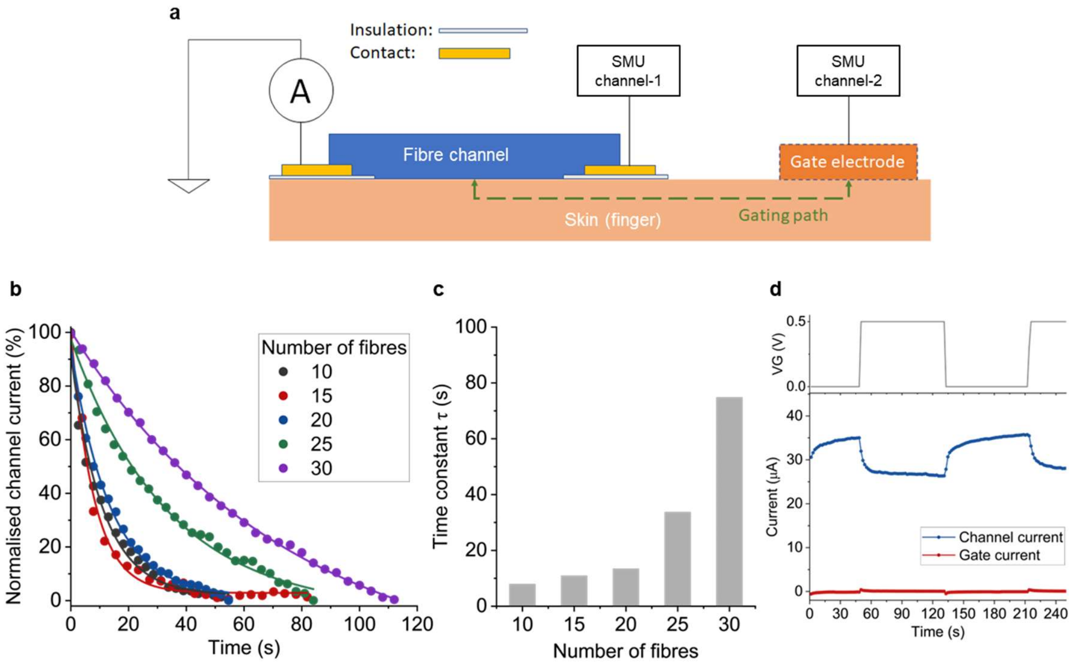

Supplementary Fig. 6 | Bioelectronic fibres as skin-gated OECT. a, Schematic drawing of the skin-gated OECT set up. **b,** OECT responses under a + 0.5 V gate voltage input versus number of fibres in the SMU channel, with exponential fitting curves. **c**, Time constants τ of the exponential fitting curves from (**b**). **d,** Channel and gate currents of the skin-gated OECT with 15 fibres as the channel.

Supplementary Note 7: Comparison of the environmental footprints between orbital spinning and microfabrication

The power consumption of the orbital fibre production mechanism is less than 2 W (Supplementary Note 1). Considering the fibre production mechanism is mounted onto a platform with translational and rotational movements, the total power consumption would not exceed 50 W (estimated based on energy consumption of two Thorlabs translational stages and a servo motor). Therefore, 1 kWh of electricity would be able to power the orbital spinning for at least 20 hours of operation, producing over 50 kilometre length of fibres. For the energy consumption of micropatterning PEDOT:PSS high resolution line features via masked electrodeposition[28–31], a number of sources need to be considered: cleanroom air treatment overhead, spin coater, mask production & patterning, lithography, mask etching, mask lift-off, thermal/e-beam evaporation. In a conservative measure, the power consumption for micropatterning in a cleanroom can easily exceed 100s of kilowatts. In this comparison, 1 kWh of electricity would be insufficient to power the micropatterning for even a minute (by average power estimation of the full patterning process). Therefore, to yield similar consumed energy per unit length of PEDOT:PSS line pattern as the orbital spinning, the micropatterning need to achieve 50 km long line patterns within a minute. Therefore, microfabrication is still ill-suited for scalable customisations.

Supplementary Note 8: Comparison of the environmental footprint between the fibre arrays in this work and the washing cycles of fabrics

According to the literature, microfibres released from washing per 1 kg of fabrics by washing machine is around 124-308 mg[32]. Thus, the washing of per 1 gram of synthetic fabrics would release around 0.124-0.308 mg of microfibres, which is equivalent to the total 0.1-0.3 mg of dry mass input for forming each fibre array device. For a typical household washing machine with 5-6 kg loading per cycle, the microfibre waste generated from one cycle of washing would be around 620-1,540 mg, equivalent to the dry mass of more than 5,000 organic bioelectronic fibre interfaces. In addition, producing 5,000 fibre arrays made from bioelectronic fibres would require at most 30 mL solution of water (considering one array is created within 5 minutes, and the solution feeding is at the rate of 1 μL per minute). In comparison, the water usage of each washing cycle by the 5-6 kg loading washing machine is around 70 litre[33]. It is to note that machine-washing is only used to upkeep the duration of the use of the textiles, while the production and disposal of traditional synthetic textiles are already a highly environmental damaging process[34].

Additional supplementary figures:

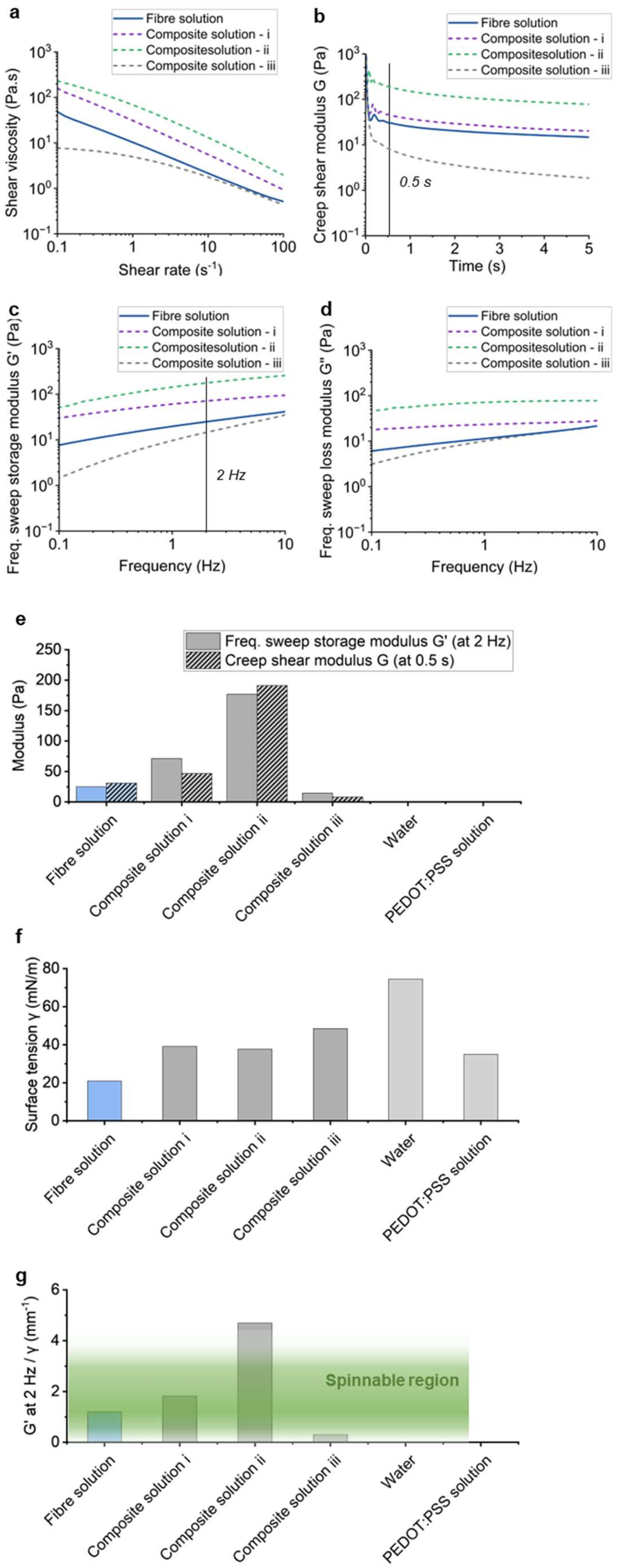

Supplementary Fig. 7 | Rheological and surface tension characterisations of the fibre solution with reference composite solutions to identify the 'spinnable' solution property region for the orbital fibre spinning technique. a, Shear viscosity obtained from steady-shear measurements. **b,** Creep shear modulus (G) extrapolated from shear creep measurements with 1 Pa of step stress applied. **c,** Storage modulus (G') and **d,** loss modulus (G'') obtained from oscillatory shear-frequency sweep measurements under 1 % of shear strain. **e,** Creep shear modulus values at 0.5 s from (**b**) and storage modulus values at 2 Hz from (**c**). **f,** Surface tension measurements. **g,** The ratio of elastic modulus (G' at 2 Hz) over surface tension. Reference composite solution i to iii are PEO (8M Da, 2 % w/w), PEO (8M Da, 2 % w/w)+HA (0.5 % w/w), and PEO (4M Da, 2 % w/w), respectively (all dissolved in water). The rheological characterisations were performed with a parallel plate configuration (plate gap distance at 1 mm) by a Kinexus KNX2112 rheometer at 25 °C.

It is to note that in the histogram shown in **Supplementary Fig. 7e**, the defined creep shear modulus (G) and storage modulus (G') values for different solution formulations correspond to the measurement frequency or time point at 2 Hz or 0.5 s, respectively, and the values appear to be similar for the same solution composition. The reason of choosing 0.5 s (2 Hz) as the time point is because during in situ fibre tethering, it takes ~ 0.5 s for the fibres to be initiated and land on a target. Thus, the lower frequency storage modulus (<< 2 Hz) or longer time creep response (>> 0.5 s) of the solution is not relevant to the fibre formation stage, and the higher frequency storage modulus (>> 2 Hz) or shorter time creep response (<< 0.5 s) will be subjected to high degrees of measurement uncertainties. However, even if different frequencies (or time points) were chosen other than 2 Hz (or 0.5 s) to measure the modulus, the ranking of modulus among the solutions stays the same and does not affect the final conclusion.

Additional discussion on fibre 'spinnability': In the orbital spinning set-up, in order for a fibre to be successfully initiated by mechanical stretching and then to span several centimetres in distance between the nozzle and the rotating arm, molecular chains within the solution must exist in a long-range, weakly percolated elastic state. Such percolation ability of the polymer chains is indicated by the elastic storage modulus (G') of the solution (*i.e.*, a bulk solution effect). Therefore, G' is considered the most appropriate indicator for characterising the fibre 'spinnability', instead of the solution's flowability (*i.e.*, shear viscosity).

The countering effect for fibre formation is the solution surface tension (γ) (*i.e.*, surface effect), which could result in beads-on-string structures or even solution fibre breakage. The ratio of G' to γ could be used as an indicator to characterise the solution 'spinnability' under this technique. If the ratio is small, surface effects of the solution (*i.e.*, surface tension) will play a more prominent role than the bulk solution effect (*i.e.*, elastic storage modulus), and the fibre will not be 'spinnable'. For example, solutions with a very low G' cannot be used to form fibres with this technique.

Taken together, the results show that the PEDOT:PSS (and its additives) and PEO components in the bioelectronic fibre solution naturally help to reduce the surface tension of the solution. Further, incorporating hyaluronic acid (HA) into long-chain polyethylene oxide (PEO) could be useful in promoting a robust chain percolation (as indicated by increased shear modulus). However, excessively strong molecular chain connection would also make the solution un-drawable likely due to elastic re-coil and hindered chain sliding. Therefore, a region of 'spinnability' was indicated in **Supplementary Fig. 7g**.

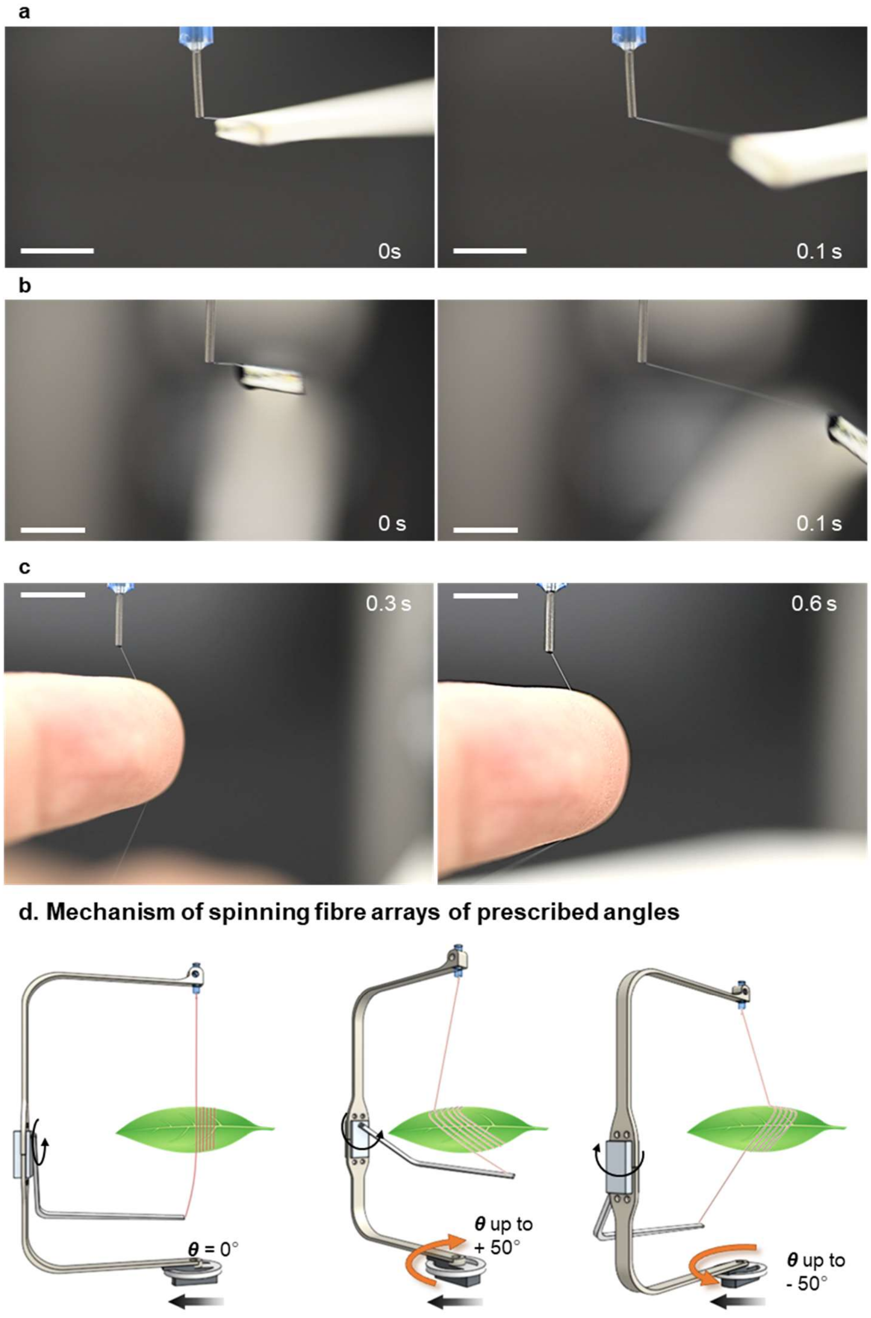

Supplementary Fig. 8 | Fibre initiation and spinning process. a-b, Fibre initiation process imaged from **(a)** side view, and **(b)** front view. The rotating arm first shear the pendent droplet from the outlet of the needle, and then the rotating movement would stretch the viscoelastic droplet to form a fibre. **c,** Photos showing the fibre deposition on an index finger. (scale bars = 5 mm). **d** Sketches showing the mechanism of spinning fibres of prescribed orientations using lab-based orbital spinning set-up. The fibre spinning platform is mounted on a servo motor that could rotate the whole platform from - 50° to + 50° to enable the tethering of fibre arrays of various angles.

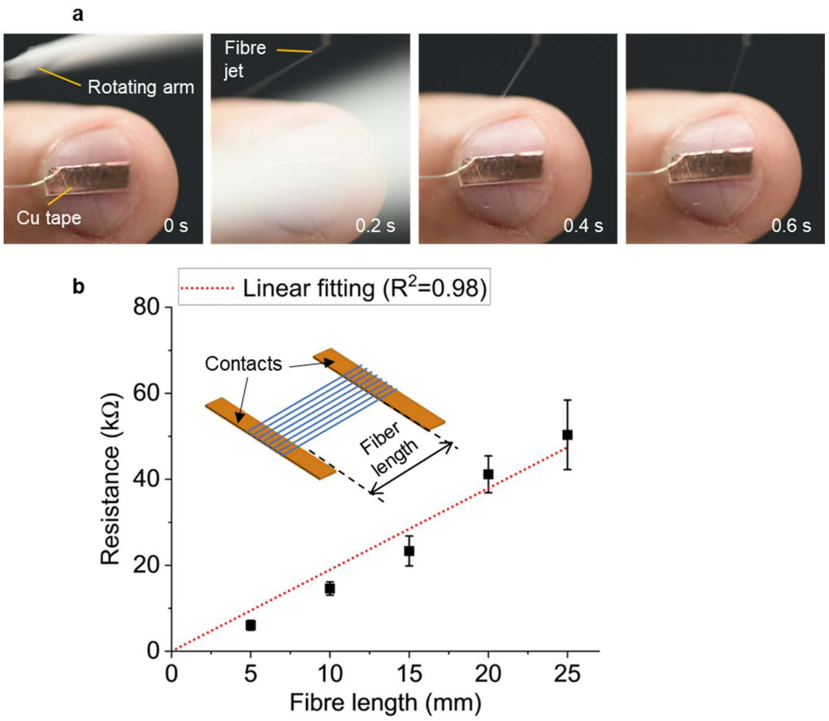

Supplementary Fig. 9 | Fibre-to-circuit electrical connections. a, Photos showing the tethered bioelectronic fibre forming direct connections on a piece of copper tape on the nail of a finger. **b**, Transmission line method (TLM) measurement to evaluate the electrical contact resistance between an array of suspended bioelectronic fibres over two copper tape contacts. The resistance of the fibre array ($\frac{N}{d} \sim \frac{50}{5\,mm}$) was measured by altering the distance between two copper electrode contacts, and the linear line fitting shows that the contact resistance is negligible compared to the axial fibre resistance (for each fibre length, 3 samples have been measured, and data are presented as mean values ± standard deviation).

Based on the TLM measurement, the conductivity of the bioelectronic fibres could be calculated as ~ 5,000 S/m, comparing similarly or favourably with other reported PEDOT:PSS fibre systems[35,36]. The resistance of the fibre array (R) could then be estimated by through $R = \frac{L}{K \cdot N}$, where L is the macroscopic fibre length, N is the number of fibres in the array, and $K \sim$ 0.0015 S·µm is an approximate constant relating to the average width of bioelectronic fibres (w) and conductivity.

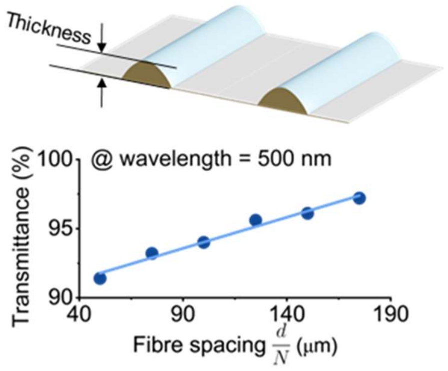

Supplementary Fig. 10 | Fibre array transmittance with various fibre spacings ($\frac{d}{N}$).

The transmittance of the fibre array is plotted versus $\frac{d}{N}$ (with d in μm) because that gives a seemingly linear relationship. The best linearly fitted equation for transmittance at 500 nm wavelength is transmittance T (%) = 0.045 $\frac{d}{N}$ + 89. In other words, due to the small fibre thickness (~ 1 μm), when the surface is approaching fully covered with a single layer of parallel fibres (*i.e.*, *N* is large), T (%) → 89 (%). Such a transmittance limit of ~ 89 % is similar to published data on the transmittance of micron-thick PEDOT:PSS films measured at 500 nm[37,38].

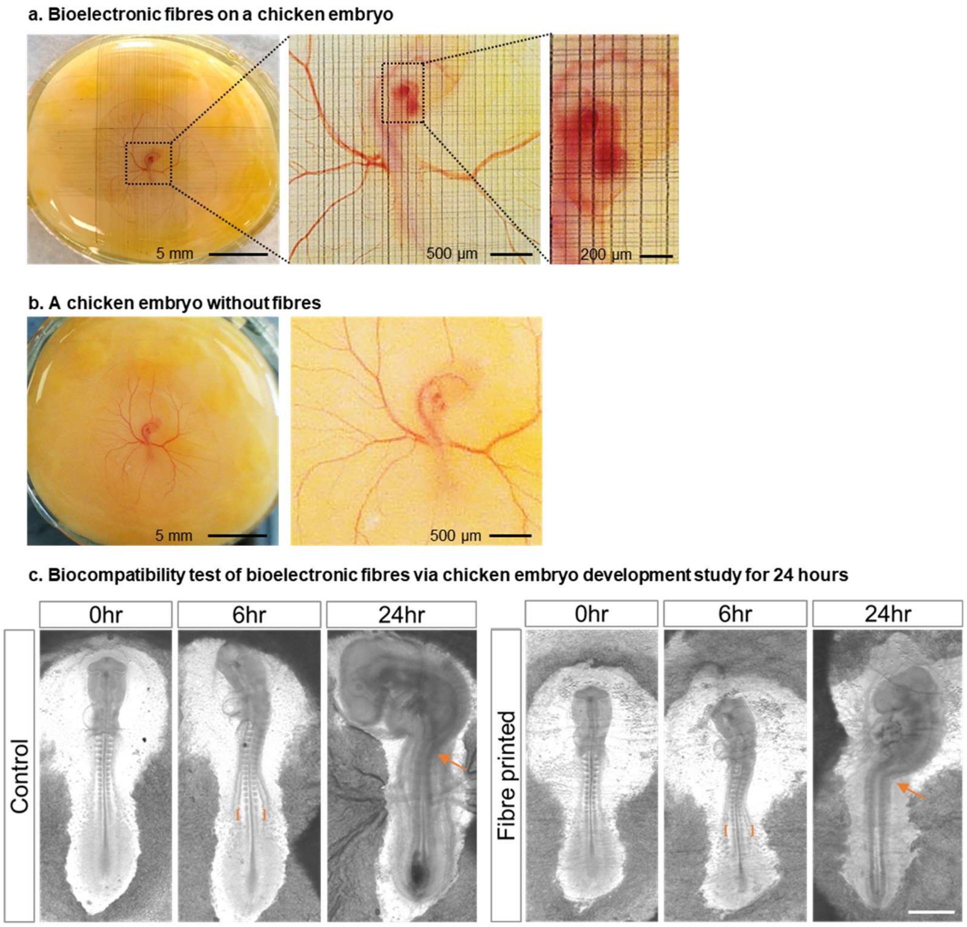

Supplementary Fig. 11 | Bioelectronic fibres interfaced with chicken embryos, and biocompatibility test. a, Magnified photos showing the bioelectronic fibres deposited on a chicken embryo. **b,** A chicken embryo without fibres. **c,** Biocompatibility test of bioelectronic fibres via chicken embryo development study for 24 hours. The embryos showed a normal rate of somitogenesis (a hallmark measure for developmental speed) at 0.63±0.08 somites per hour (n=4), which is indistinguishable from control groups without fibres (0.62±0.07, n=5). In addition, the embryos showed normal axis turning, as indicated by the thoracic flexure and turning of the brain, and developed largely normal morphology at 24 hours post printing. Fibres were patterned at a spacing of ~ 100 µm all over the surface of the chicken embryo, but due to the small fibre sizes, they are not clearly visible under the specified microscopic setting. (The orange brackets indicate newly formed somites and the orange arrows indicate thoracic flexure, scale bar = 1 mm).

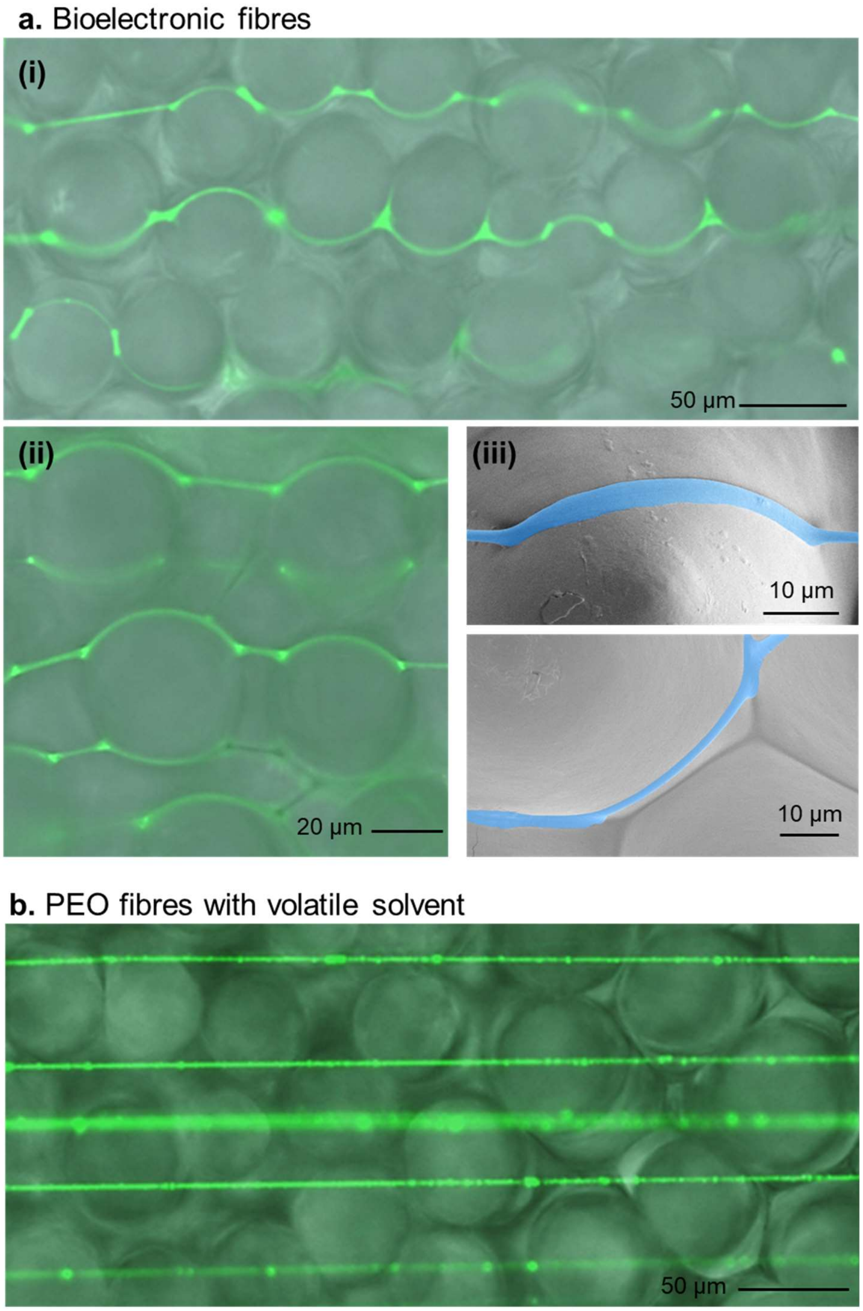

Supplementary Fig. 12 | Images showing the fibre tethering contacts on orchid flower petals for **a,** bioelectronic fibres doped with a fluorescent dye (**i** and **ii** are fluorescent optical microscopic images, **iii** is an SEM image with false colouring for the bioelectronic fibres) and **b,** PEO fibres (optical microscopic image) under prepared with a volatile solvent (2 % 8 M Da PEO dissolved in 80 % v/v ethanol). It could be seen from the images that the bioelectronic fibres morph onto the topographies of the flower petal surfaces; while the PEO fibres produced with a volatile solvent suspend on top of the surface.

For the optical microscopic imaging, in order for clear visualisation of the fibres, Fluorescein Sodium (20 µg/mL, Sigma-Aldrich) was added to the fibre solutions. The optical images were overlaid with the FITC channel (showing the fibres) and the brightfield view.

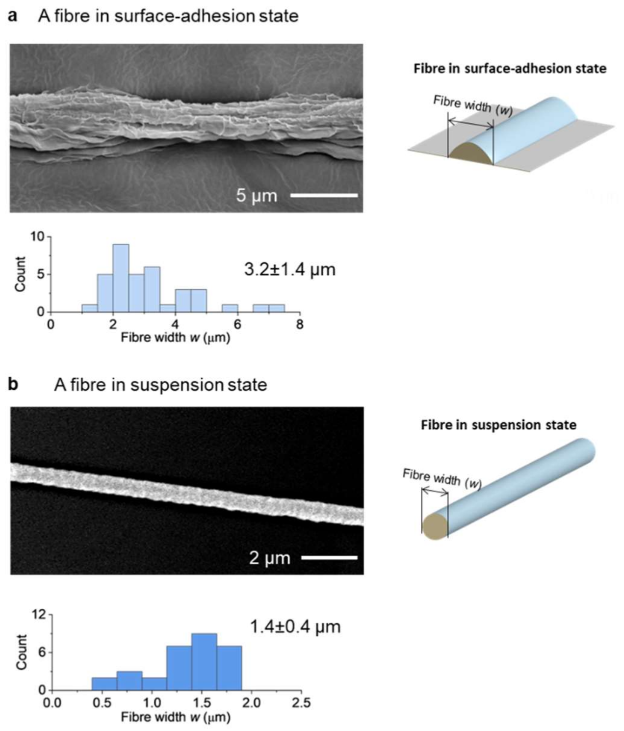

Supplementary Fig. 13 | SEM images and histograms showing fibre feature width (*w*) of bioelectronic and pH-responsive fibres in (a) surface-adhesion, and (b) suspension states. The feature size, or width (*w*), of the deposited fibres would be affected by the contact states. For fibres in the surface-adhesion state, the cross-sectional shape would become semi-circular, and the average fibre width is 3.2±1.4 µm. This is because the freshly initiated fibre remains semi-wet when deposited onto the surface of the target object and the surface wetting would result in the fibre spreading out. Suspended fibres would usually have a circular cross-sectional shape[39], and the average diameter is 1.4±0.4 µm.

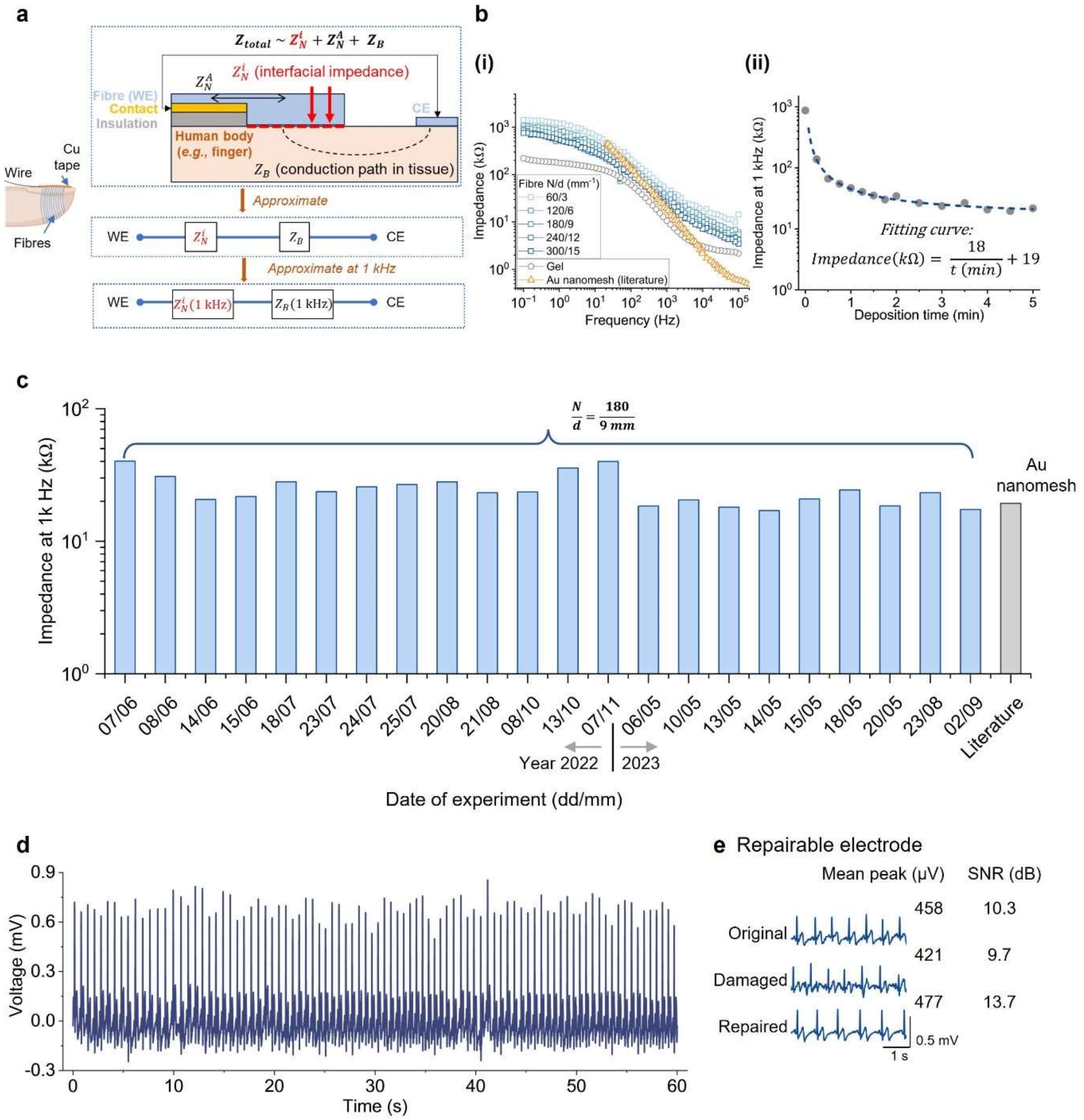

Supplementary Fig. 14 | Bioelectronic fibres on a fingertip for biopotential monitoring. a, Schematic showing the connection design between bioelectronic fibres with the outer circuit on index fingers for contact impedance and ECG measurements, with a simplified diagram to model the overall impedance measured for bioelectronic fibre electrode on skin ("WE" is working electrode, "CE" is counter electrode.). **b, (i)** a typical impedance spectrum showing the contact impedance on the index finger with respect to fibre deposition time, and in comparison with a microfabricated gold nanomesh electrode[4] and a gel electrode. **(ii)** Impedance value at 1 kHz versus deposition time with a fitting curve (see explanations of the fitting as below). **c,** Skin contact impedances of bioelectronic fibre electrodes deposited on an index finger measured on different days. It is shown that the fibres produced on different dates of experiments have a consistent level of contact impedance, comparable to a microfabricated gold nanomesh[4]. **d,** A typical continuous 1 minute ECG measurement with a bioelectronic fibre array from the fingertip. **e,** ECG signals measured from the original, damaged, and repaired fibre electrode (the mean peak value is calculated from the mean R peaks in the ECG signals).

We could model the bioelectronic fibre electrode on skin as a simplified circuit shown in the above Fig. a. The impedance/resistance of the entire circuit: $Z_{total} \sim Z_N^i + Z_N^A + Z_B$, where Z_N^i is the fibre array skin/contact interfacial impedance, Z_N^A is the fibre array axial impedance, and Z_B is the impedance of bulk skin and body.

Since for N~180, DC axial resistance of the fibre array around the entire fingertip is estimated to be ~5 kΩ (c.f. Supplementary-Table 2 shows 10 kΩ for N~100), while Fig. b(i) above shows Z_{total} (f=0.1Hz) ~ 10^3 kΩ, thus in this setup, $Z_N^A \ll Z_N^i + Z_B$ (where Z_N^A corresponds to only the ~mm distance between the metal contact and where the fibres start to tether the skin).

Therefore, we can assume $Z_{total} \sim Z_N^i + Z_B$,

For explaining the relationship between the impedance Z_{total}(f=1kHz) versus deposition time and the fitting curve in Fig. b(ii) above,

$$Z_N^i \text{ (1kHz)} \sim \frac{\zeta}{AN} = \frac{\zeta}{Akt}$$

Where N is the number of fibres (N=kt, k is the fibre deposition rate, and t is the deposition time), A is the typical area of contact between each fibre and skin, ζ is the typical impedance of a single fibre at the interfacial layer.

$$\text{Thus, } Z_N^i \text{ (1kHz)} \sim \frac{C_1}{N} \sim \frac{C_2}{t}$$

where C_1 & C_2 are approximate constants

Thus, Z_{total}(1kHz)~ $\frac{C_1}{N} + Z_B$ or Z_{total}(1kHz)~ $\frac{C_2}{t} + Z_B$ can be the form of the fitted line. In this experiment, the fitted line equation is Z_{total}(in kΩ unit) $= \frac{18}{t} + 19$.

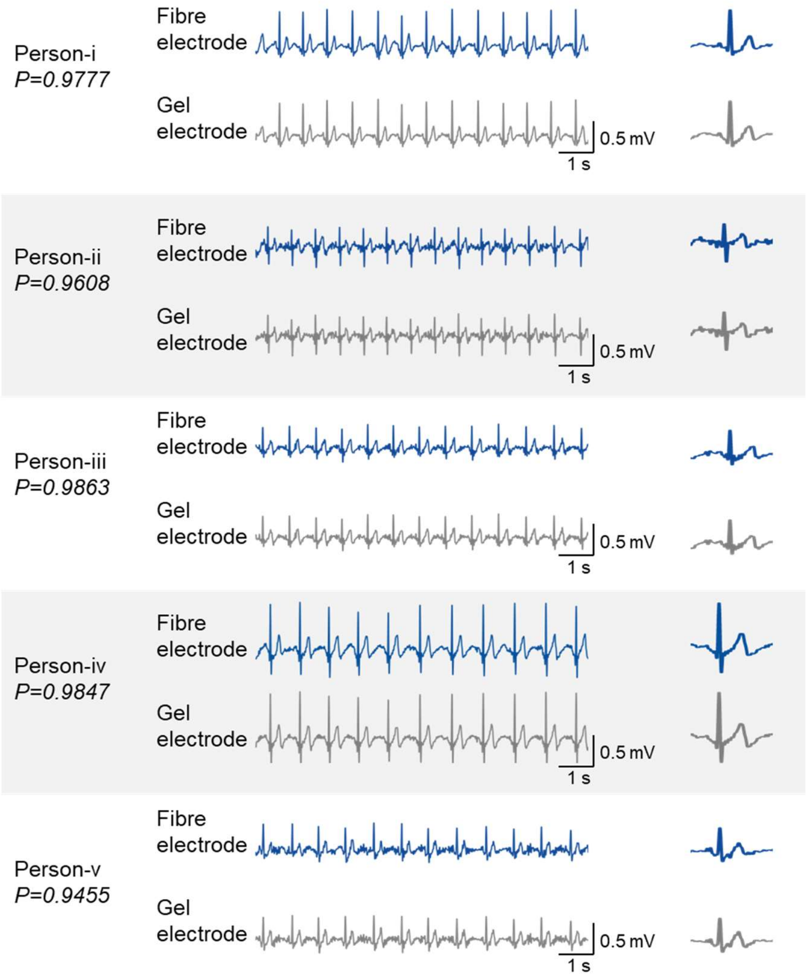

Supplementary Fig. 15 | Comparing simultaneous ECG measurements from a fibre or a gel electrode for five volunteers (*P* represents data correlations*). During the measurements, the fibre electrode was deposited onto the fingertip of the index finger while the gel electrode was attached onto the fingertip of the middle finger of the same person, and the signals were measured at the same time.

*the data correlation *P* represents Pearson correlation coefficient[40], which is usually used to compare the ECG signals acquired simultaneously with lab-produced devices and standard commercial electrodes (e.g., hydrogel electrodes)[41–43]. In this work, the coefficient was calculated by a standard MATLAB algorithm[44].

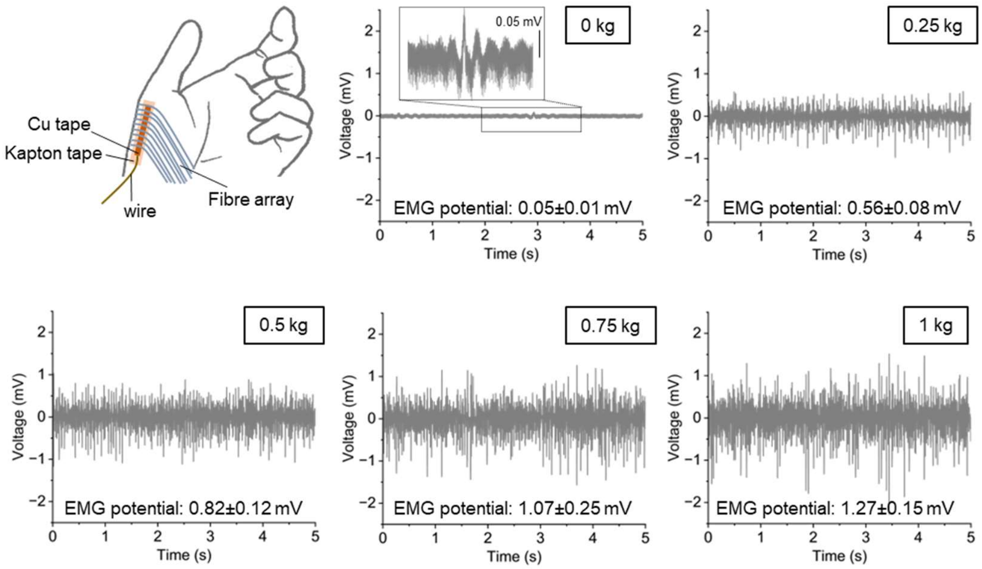

Supplementary Fig. 16 | Variations of the muscle EMG amplitude versus the weight of loading on hand. Bioelectronic fibres were deposited onto the thumb-thenar muscle area, and loadings of various weights were applied onto the thumb. (Typical results from N=5 volunteers, with n>3 independent experiments performed on each volunteer).

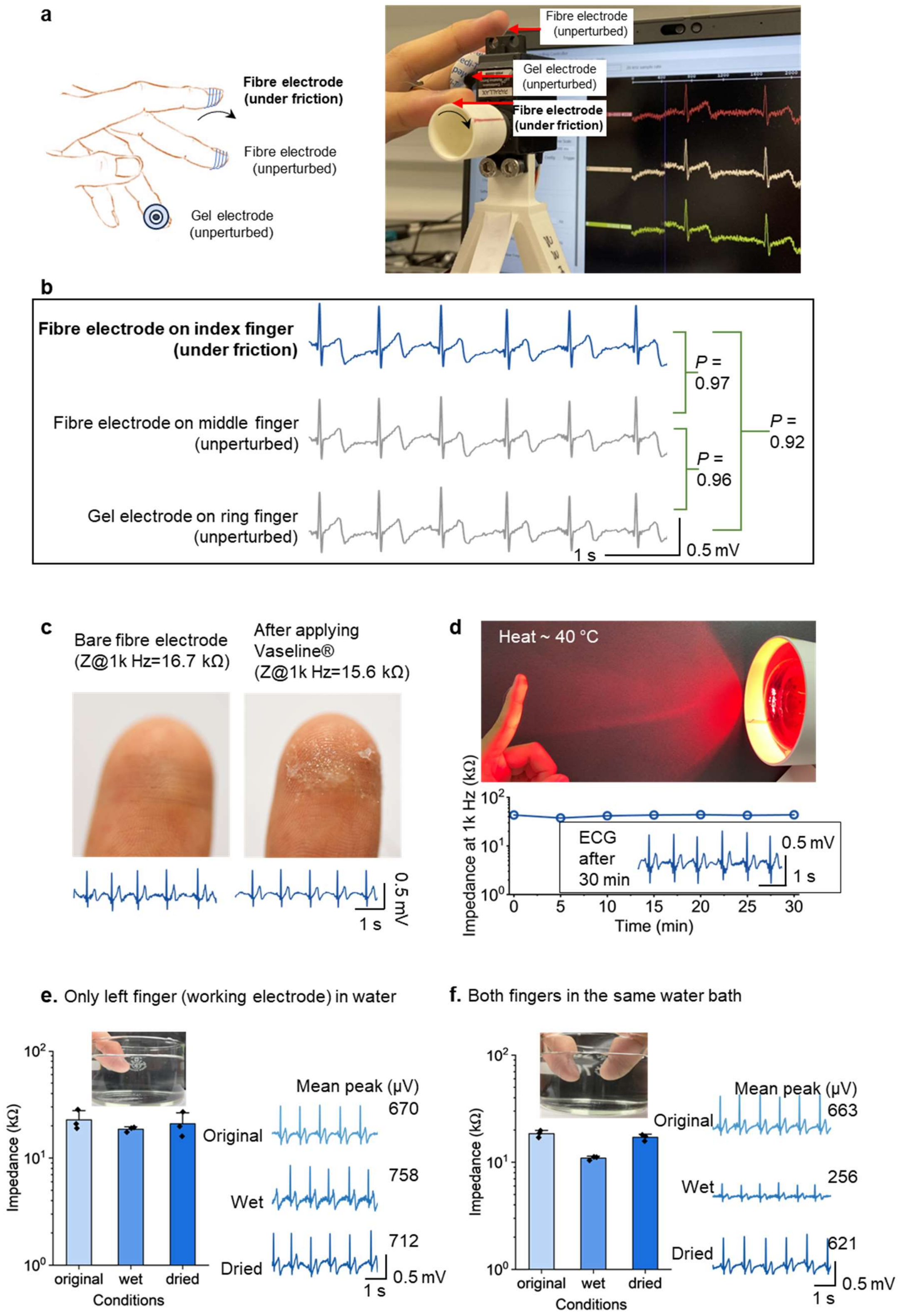

Supplementary Fig. 17 | The stability of bioelectronic fibres for biopotential monitoring during dry frictions, with simulated grease, or under heat, and water-soaking conditions. **a,** Schematic and image illustrations showing the experimental design for verifying the effect

of dry surface friction on the ECG signal acquisition. Bioelectronic fibre electrodes were tethered on the index and middle fingers, and a commercial hydrogel electrode was worn on the ring finger of the same hand. Only the fibre electrode on the index finger was subjected to friction with a polylactic acid plastic drum (surface friction speed ~ 4 cm/s and normal force ~ 2 N). **b,** ECG signals collected simultaneously from the three electrodes on fingers, with Pearson correlation coefficients (*P*) comparing the signal correlations between the fibre electrode subjected to friction and the other unperturbed electrode. **c,** Vaseline® is applied on the bioelectronic fibre electrode to simulate the effect of grease. **d,** The fingertip with bioelectronic fibre electrode was placed ~ 30 cm in front of an 100 watt infrared lamp (~ 40 °C). **e,** Contact impedance and ECG sensing performance negligibly change when only left index finger (working electrode) is soaked in water, and dried after removal from water. **f,** Contact impedance and ECG sensing performance when both left (working electrode) and right (counter electrode) index fingers are soaked in the same water bath. (The mean peak value is calculated from the mean R peaks in the ECG signals. For impedance values, data are presented as mean values ± standard deviation, and 3 independent measurements have been conducted for each condition).

It is seen in **e** that when both working and counter fibre electrodes are soaked in the same water bath, the surface impedance and ECG amplitude decrease evidently (but still clearly visible). This is because the water bath 'short-circuit' the working and counter electrodes (the current loss between the working and counter electrodes via the water bath). This test demonstrates that the fibre electrodes could fully replace the gel electrodes for biopotential sensing, and ECG sensing could still carry out even when the exposed fibre electrodes are 'short-circuited'. Nonetheless, the performance retained after the fingers being removed from the water bath and dried.

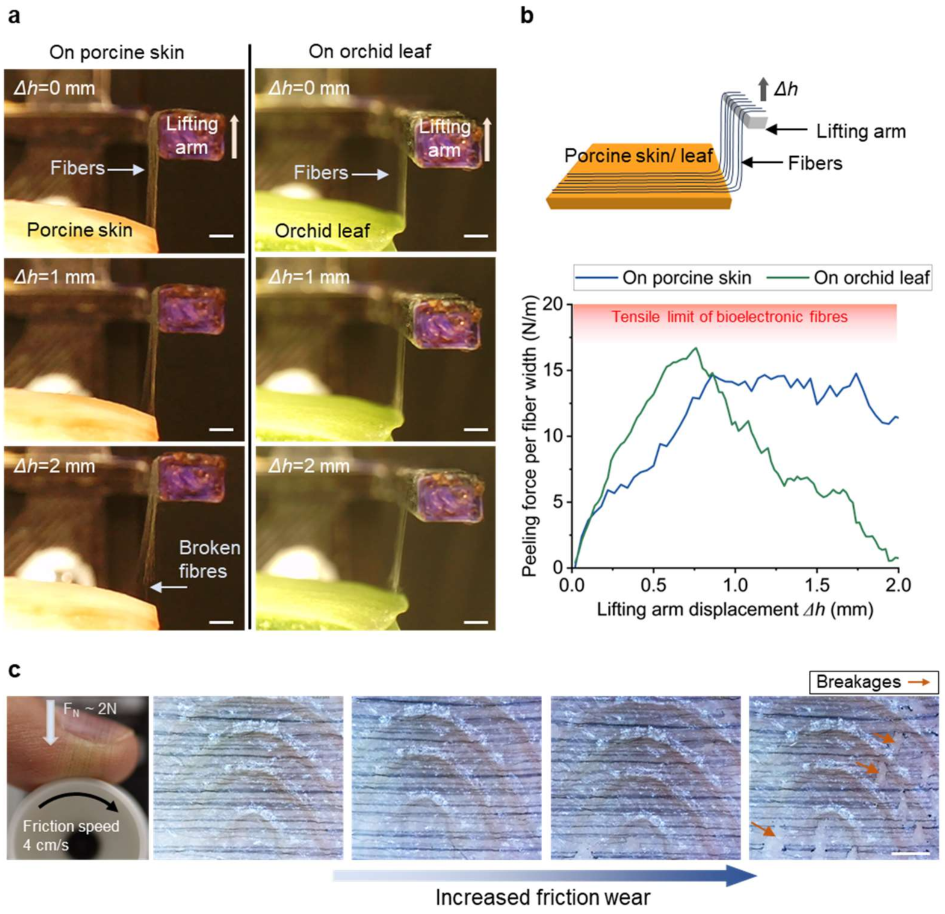

Supplementary Fig. 18 | Surface adhesion characterisations and failure mechanisms of the bioelectronic fibre arrays on typical living structure surfaces under dry conditions. a, The process of a modified 90-degree peeling test (derived from the ASTM D2861 90-degree peeling test): an array of bioelectronic fibres ($\frac{\mathrm{N}}{\mathrm{d}} \sim \frac{200}{10\ \mathrm{mm}}$) being peeled off from a piece of porcine skin or an orchid leaf (scale bars, 2 mm). The fibre number density of $\frac{\mathrm{N}}{\mathrm{d}} \sim \frac{200}{10\ \mathrm{mm}}$ (thus average fibre spacing $\frac{\mathrm{d}}{\mathrm{N}} \sim 50\ \mu\mathrm{m}$) was used in the peeling test because it resembles the fibre number density used for on-skin biopotential monitoring (*i.e.*, $\frac{\mathrm{N}}{\mathrm{d}} = \frac{180}{9\ \mathrm{mm}}$ for ECG sensing from a fingertip). **b,** Peeling force per unit fibre width measured from the peeling experiment (estimated through $\frac{\mathrm{F}}{3\times10^{-6}\cdot\mathrm{N}}$, where *F* is the peeling force in Newtons, *N* is the number of fibres deposited, and 3×10^{-6}m (or 3 μm) is the assumed mean width of each bioelectronic fibre, *w*). **c,** Microscopic images showing the local shear fractures of a bioelectronic fibre array on a fingertip undergoing excessive dry frictions (scale bar, 500 μm).

<u>Additional discussion on the adhesion properties of the bioelectronic fibres</u>: the maximum recorded peeling force per fibre width is ~ 15 N/m in both cases, which are comparable to hydrogel and film-based skin electronics from literature[2,45–48]. The photos in Supplementary Fig. 18a show that the peeling process ultimately resulted fibre breakage from the skin models instead of noticeable delamination, when the peeling force is approaching the bioelectronic fibres' tensile limit (~ 20 N/m under dry ambient room conditions, Supplementary Fig. 5).

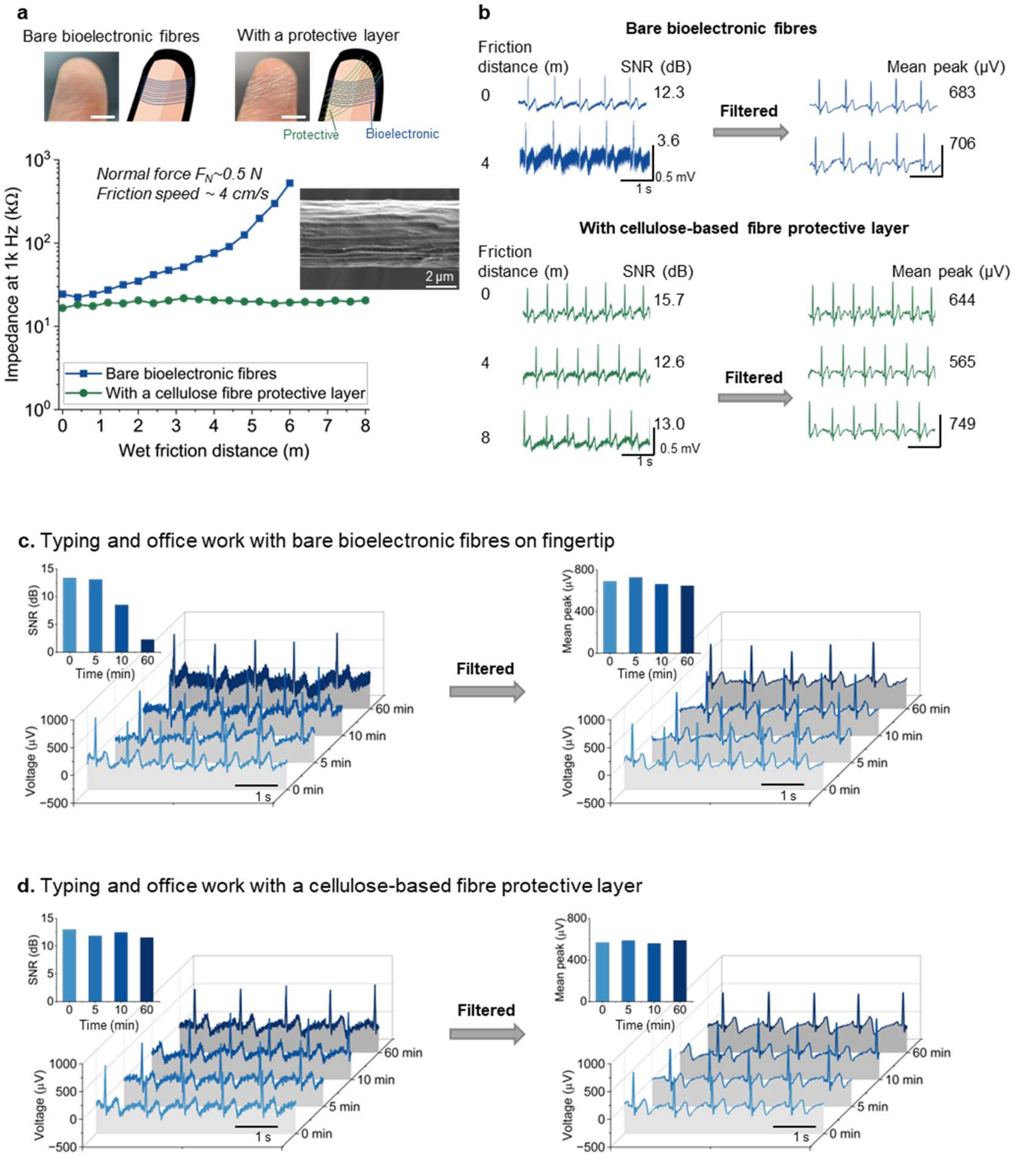

Supplementary Fig. 19 | The effect of environmental disturbance on the stability of bioelectronic fibres and strategies to mitigate the effect. a, A protective layer of cellulose-based fibres ($\frac{N}{d} \sim \frac{90}{9\,mm}$) could be tethered on top of the bioelectronic fibre array at a tilted orientation ($\Delta\theta \sim 30°$) to enhance the stability of bioelectronic fibres against wet frictions (scale bars, 5 mm). The insert SEM image shows a typical cellulose-based fibre. **b,** ECG sensing performance under wet frictions with bare bioelectronic fibres on the fingertip versus with a layer of cellulose-based protective fibres. **c-d,** ECG signals measured from: **(c)** bare fibre electrodes on fingertips after performing normal typing and office work on a computer for 5, 10 and 60 minutes, and **(d)** with a layer of cellulose-based protective fibres.

It is to note that in the 'wet' friction situations where the SNR decreased over friction distance and time, application of a bandpass filter (0.5-50 Hz) onto the original ECG signals will lead to clear ECG traces with no degradation of the ECG R peak values.

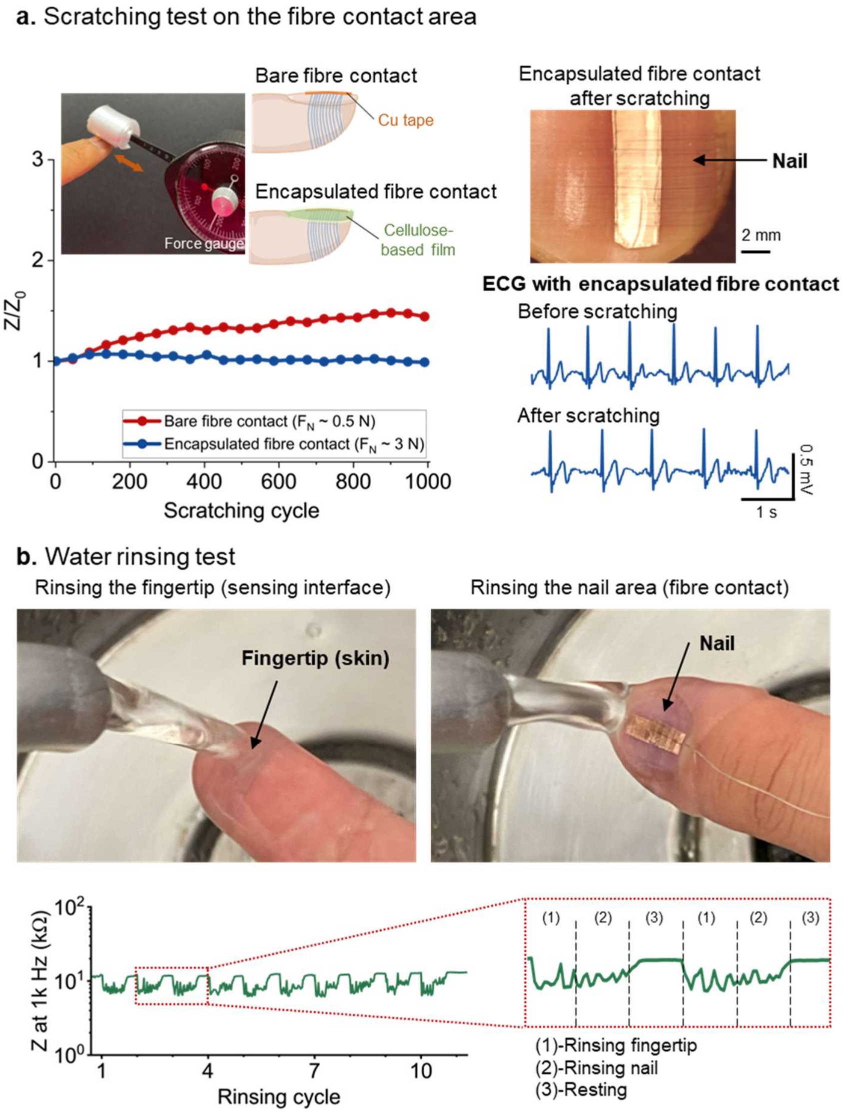

Supplementary Fig. 20 | Scratching and water rinsing tests for fibre electrodes with encapsulated fibre-to-circuit connections. a, Scratching test against fabrics performed on the fibre-circuit connection area on top of the nail, comparing bare and encapsulated fibre contacts. **b,** Water rinsing test cycles.

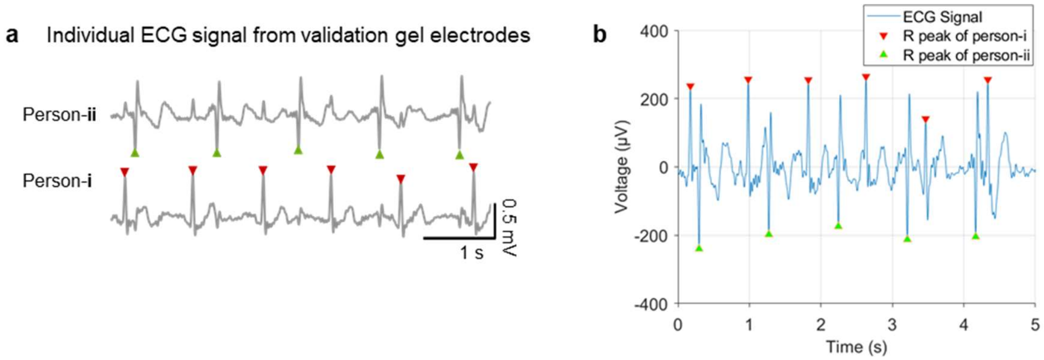

Supplementary Fig. 21 | Identifying individual ECG signals from dual-ECG sensing. a, Individual ECG signals measured with gel electrodes for validation. **b,** The results from a MATLAB program to identify the R peaks for both people from the dual-ECG signals measured by fibre electrode (The peaks were identified by directly using the ‘findpeaks’ function in MATLAB, with setting the peak value threshold as ±100 μV and peak spacing as 0.5 s).

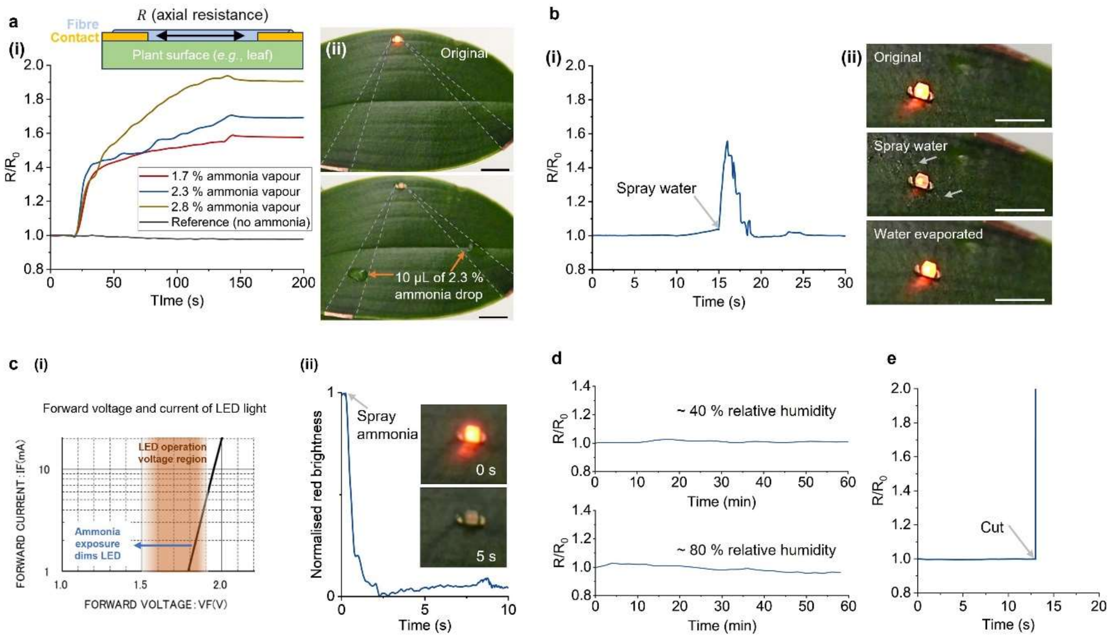

Supplementary Fig. 22 | Bioelectronic fibres as plant interfacing sensor arrays. a, (i) Ammonia vapour causing irreversible resistance increase of bioelectronic fibre arrays ($\frac{N}{d} \sim \frac{75}{3\,mm}$) (the vapours were applied at 20 s and stopped at 150 s); the inset shows the common standard schematic for axial fibre resistance measurement. **(ii)** Photos showing a control experiment with direct ammonia droplet deposition (two 10 μL droplets of 2.3 % ammonia) onto the fibre arrays on an orchid leaf, where LED dims when droplet contacts (Scale bars = 5 mm). **b, (i)** Normalised resistance of the bioelectronic fibre arrays upon water spray, but the resistance returns to the original value after water evaporation. **(ii)** Photos showing the reversible dimming of an LED light connected by bioelectronic fibres on an orchid plant leaf to the spray of water (Scale bars = 5 mm). **c, (i)** LED manufacturer's data sheet showing forward voltage *vs.* current of the LED light used in the experiment[49], with the shading to indicate the threshold voltage region of operation. **(ii)** Normalised red channel brightness of the LED light upon ammonia exposure (the red brightness was analysed by MATLAB from the video recording of a chosen field of view, as indicated by the inserted photos). **d,** The normalised resistance of bioelectronic fibres under ~ 40 % and ~ 80 % relative humidity environments. **e,** Normalised resistance when cutting an array of bioelectronic fibres.

In addition, although the bioelectronic fibres were used as conductors to connect to the micro-LED in **(a)** and **(b)**, the primary function is for sensing and a warning system. It is to note that the signal patterns of **(b)**, **(d)**, and **(e)** differ from **(a)**. Therefore, in the future it could be possible to use machine learning to distinguish the different signal patterns to improve the readout performance.

<u>Additional discussion</u>

The operational strategy for the external driving voltage of the fibre-LED chemiresistive ammonia warning system: Prior to ammonia exposure, the driving voltage ($V^* \sim 5 - 13$ V) from the power supply at the whole circuitry level is adjusted into a threshold level, which is just sufficient to make the LED ON (light up) visibly to the naked eye. Then, ammonia exposures leading to fibre resistance increase could decrease the voltage across the LED light

beyond its threshold region, causing an ON-OFF switch. In practice, if the reconfigured fibre path changes in length, customising N in the fibre array could make sure V^* does not exceed ~ 13 V to power the LED in the threshold status.

According to the manufacturer's data sheet, a voltage decrease from ~ 1.9 ± 0.1 V to ~ 1.7 V across the LED could give it a noticeable ON to OFF switch (**c-i**). In other words, when the LED light is powered at its threshold LED ON status, ~ 0.2 V reduction in voltage across the LED would be sufficient to cause the ON-off switch (**c-ii**).

In terms of the detection limit, as shown in **a-i**, ammonia exposure (as low as ~ 1.7 % in water) could cause over 40 % increase in fibre resistance ($\frac{R}{R_0} > 1.4$, where R_0 is the fibre array resistance prior to ammonia exposure). In this case, the LED threshold turn-off could happen with minimal V^* ~ 2.4 V (*i.e.*, [pre-exposure] V* = 1.9 V (LED) + 0.5 V (fibre), *versus* [post-exposure] V^* = 1.7 V (LED) + 0.5 V × 1.4 (fibre)). In the experiments, N could always be customised to tune the fibre array resistance R so that the driving voltage V^* was above 5 V, but smaller than 13 V (the upper bound is set to avoid high voltages and also considering electric field working range determined in Supplementary Fig. 23).

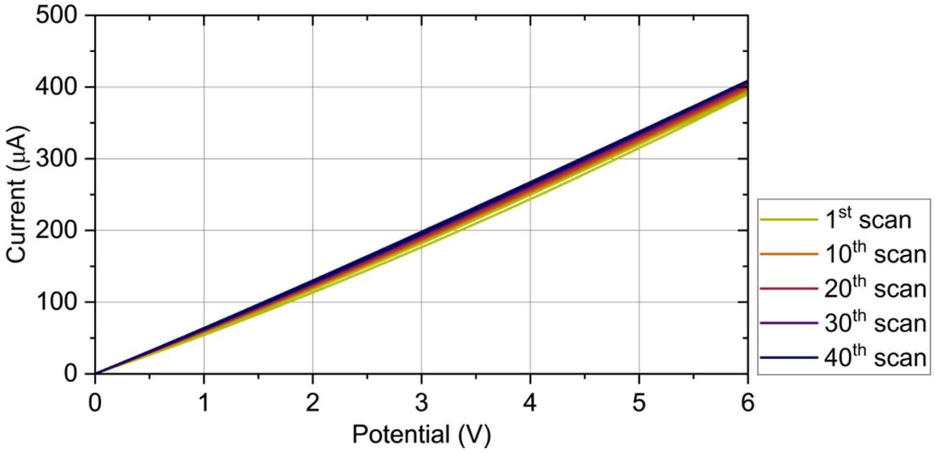

Supplementary Fig. 23 | Cyclic voltammetry measurements of bioelectronic fibre arrays. Cyclic voltammetry measurements on an array of 1 cm length bioelectronic fibres ($\frac{N}{d} = \frac{50}{5\,mm}$) deposited on a glass slide for 40 scans (scan rate at 0.2 V/s). The fibre array shows stable ohmic behaviour under 6 V/cm applied voltage.

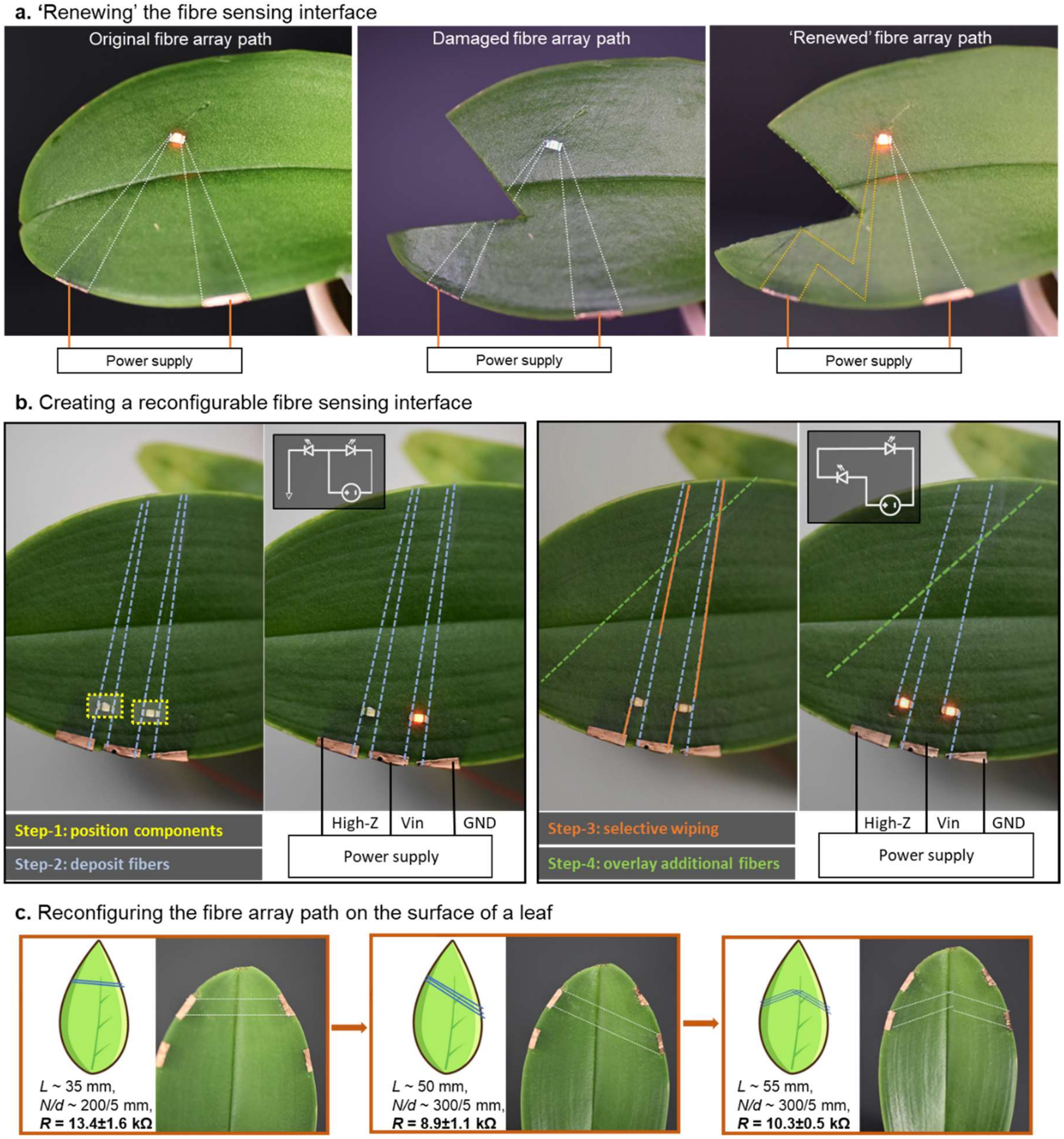

Supplementary Fig. 24 | Reconfiguring bioelectronic fibre sensing interface. a, The fibre sensing interface could be 'renewed' by re-routing the fibre arrays when the leaf is damaged, while keeping the contacts and LED light positions (the dotted lines indicate the counters of the bioelectronic fibre arrays). **b,** The steps of creating reconfigurable fibre sensing interface on an orchid plant leaf. Step-1: surfaced mounted LEDs are placed onto the leaf. Step-2: Initial four fibre arrays (in blue colour) are deposited to individually connect the four electrodes of the LEDs, and at this point, an initial sensing fibre patterning is being formed, where the two LEDs could be individually powered. Step-3: in order to reconfigure the fibre path so that both LEDs could be powered in series, unwanted fibre traces could be wiped off (in red colour). Step-4: new fibre arrays (in green colour) could be deposited to complete the reconfiguration. **c.** The fibre array on a leaf surface being reconfigured with increased path length and number of fibres in the array (NB. The electrode to electrode resistance measured on the bare leaf surface without fibres is above MΩ).

Addition notes on overall fibre path resistance and operation voltage after reconfiguration: the overall fibre path resistance R is associated with the fibre array formed by N fibres of fixed length connected in parallel format; thus, theoretically, $R \propto \frac{1}{N}$. Circuit reconfiguration could change the fibre path length (thus L). However, at the circuitry level, when the reconfigured fibre path differs in length L, the circuit level fibre array resistance R could be compensated by customizing the number of fibres N.

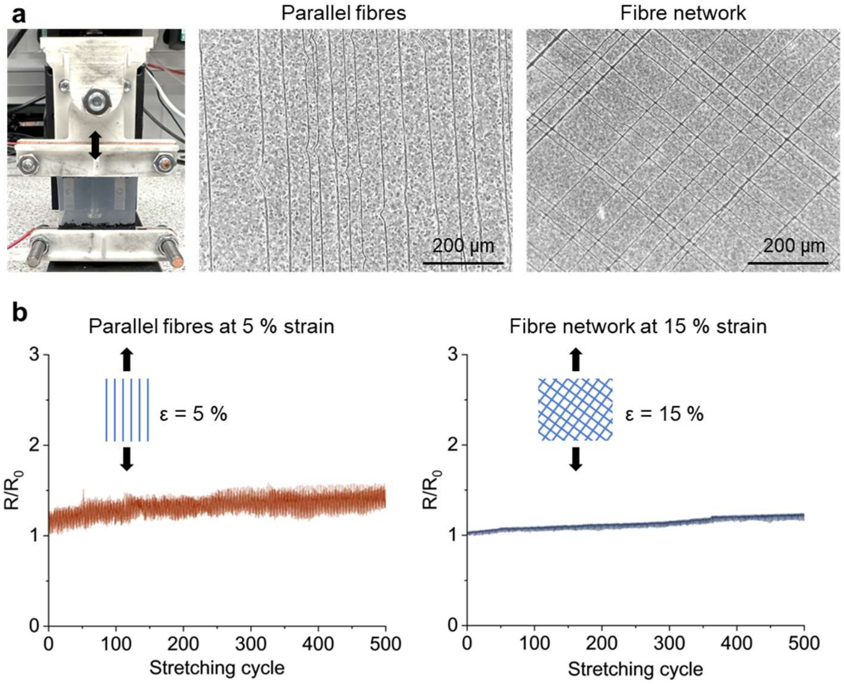

Supplementary Fig. 25 | Bioelectronic fibre network stretchability characterisations. a, A photo showing the stretching experiment with bioelectronic fibres deposited on an elastomer film (Ecoflex 00-30), and microscopic images showing the parallel and orthogonal fibre network configurations. Ecoflex elastomer was used as the substrate because it possesses high stretchability[50] and could be regarded as a 'skin-like' material[51]. Parallel fibres ($\frac{N}{d} \sim \frac{400}{2\ cm}$) and orthogonal fibre networks ($\frac{N}{d} \sim \frac{600}{3\ cm}$ in each direction) were deposited on elastomer substrates (~ 1 mm thickness, Ecoflex 00-30). A lab-designed tensile rig was assembled with 3D printed clamps attached onto a motorised stage (Thorlabs MTS50-Z8), and a multimeter (Keysight 34465A) was used to record the resistance. **b,** Cyclic stretching tests for parallel fibres and fibre networks.

References:

1. Allen, R. D. Mechanism of the Seismonastic Reaction in *Mimosa pudica*. *Plant Physiol.* **44**, 1101–1107 (1969).
2. Jiang, Z. *et al.* A 1.3-micrometre-thick elastic conductor for seamless on-skin and implantable sensors. *Nat. Electron.* **5**, 784–793 (2022).
3. Lee, S. *et al.* Nanomesh pressure sensor for monitoring finger manipulation without sensory interference. *Science* **370**, 966–970 (2020).
4. Miyamoto, A. *et al.* Inflammation-free, gas-permeable, lightweight, stretchable on-skin electronics with nanomeshes. *Nat. Nanotechnol.* **12**, 907–913 (2017).
5. Matsuhisa, N. *et al.* Printable elastic conductors by in situ formation of silver nanoparticles from silver flakes. *Nat. Mater.* **16**, 834–840 (2017).
6. Zhu, Z. *et al.* 3D Printed Functional and Biological Materials on Moving Freeform Surfaces. *Adv. Mater.* **30**, 1707495 (2018).
7. Kim, K. K. *et al.* A substrate-less nanomesh receptor with meta-learning for rapid hand task recognition. *Nat. Electron.* **6**, 64–75 (2022).
8. Chang, H. *et al.* Recreating the heart's helical structure-function relationship with focused rotary jet spinning. *Science* **377**, 180–185 (2022).
9. Qin, Z., Compton, B. G., Lewis, J. A. & Buehler, M. J. Structural optimization of 3D-printed synthetic spider webs for high strength. *Nat. Commun.* **6**, 7038 (2015).
10. Yan, W. *et al.* Single fibre enables acoustic fabrics via nanometre-scale vibrations. *Nature* **603**, 616–623 (2022).
11. Al-Shorman, M. Y., Al-Kofahi, M. M. & Al-Kofahi, O. M. A practical microwatt-meter for electrical energy measurement in programmable devices. *Meas. Control* **51**, 383–395 (2018).
12. Hecker, L. *et al.* Guided Assembly and Patterning of Intrinsically Fluorescent Amyloid Fibers with Long-Range Order. *Nano Lett.* **21**, 938–945 (2021).
13. Wenzel, R. N. Resistance of solid surfaces to wetting by water. in *Industrial & Engineering Chemistry* vol. 28(8) 988–994 (1936).
14. Israelachvili, J. The different faces of poly(ethylene glycol). *Proc. Natl. Acad. Sci.* **94**, 8378–8379 (1997).
15. Hirabayashi, T. *et al.* PNPLA1 has a crucial role in skin barrier function by directing acylceramide biosynthesis. *Nat. Commun.* **8**, 14609 (2017).
16. Yan, Z. *et al.* Highly stretchable van der Waals thin films for adaptable and breathable electronic membranes. *Science* **375**, 852–859 (2022).
17. Rivnay, J. *et al.* Organic electrochemical transistors. *Nat. Rev. Mater.* **3**, 17086 (2018).
18. Khodagholy, D. *et al.* High transconductance organic electrochemical transistors. *Nat. Commun.* **4**, 2133 (2013).
19. Yang, S. Y. *et al.* Electrochemical transistors with ionic liquids for enzymatic sensing. *Chem. Commun.* **46**, 7972 (2010).
20. Qassem, M. & Kyriacou, P. Review of Modern Techniques for the Assessment of Skin Hydration. *Cosmetics* **6**, 19 (2019).
21. Logan, R. W. & McClung, C. A. Rhythms of life: circadian disruption and brain disorders across the lifespan. *Nat. Rev. Neurosci.* **20**, 49–65 (2019).
22. Shannon, R. V. A model of safe levels for electrical stimulation. *IEEE Trans. Biomed. Eng.* **39**, 424–426 (1992).
23. Groiss, S. J., Wojtecki, L., Südmeyer, M. & Schnitzler, A. Review: Deep brain stimulation in Parkinson's disease. *Ther. Adv. Neurol. Disord.* **2**, 379–391 (2009).
24. Trout, M. A., Harrison, A. T., Brinton, M. R. & George, J. A. A portable, programmable, multichannel stimulator with high compliance voltage for noninvasive neural stimulation of motor and sensory nerves in humans. *Sci. Rep.* **13**, 3469 (2023).
25. Landgraf, B. TENS unit buyer's guide: how to choose a TENS machine. https://carex.com/blogs/resources/tens-unit-buyer-s-guide-how-to-choose-a-tens-machine#:~:text=Most%20TENS%20units%20provide%20up,intensity%20needed%20to%20block%20it (2023).

26. Rivnay, J. *et al.* High-performance transistors for bioelectronics through tuning of channel thickness. *Sci. Adv.* **1**, e1400251 (2015).
27. Yang, A. *et al.* Wearable Organic Electrochemical Transistor Array for Skin-Surface Electrocardiogram Mapping Above a Human Heart. *Adv. Funct. Mater.* **33**, 2215037 (2023).
28. Loomans, M. G. L. C., Molenaar, P. C. A., Kort, H. S. M. & Joosten, P. H. J. Energy demand reduction in pharmaceutical cleanrooms through optimization of ventilation. *Energy Build.* **202**, 109346 (2019).
29. Kircher, K., Shi, X., Patil, S. & Zhang, K. M. Cleanroom energy efficiency strategies: Modeling and simulation. *Energy Build.* **42**, 282–289 (2010).
30. Zendehdel, M., Yaghoobi Nia, N., Paci, B., Generosi, A. & Di Carlo, A. Zero-Waste Scalable Blade–Spin Coating as Universal Approach for Layer-by-Layer Deposition of 3D/2D Perovskite Films in High-Efficiency Perovskite Solar Modules. *Sol. RRL* **6**, 2100637 (2022).
31. Koutsouras, D. A. *et al.* Impedance Spectroscopy of Spin-Cast and Electrochemically Deposited PEDOT:PSS Films on Microfabricated Electrodes with Various Areas. *ChemElectroChem* **4**, 2321–2327 (2017).
32. De Falco, F., Di Pace, E., Cocca, M. & Avella, M. The contribution of washing processes of synthetic clothes to microplastic pollution. *Sci. Rep.* **9**, 6633 (2019).
33. Katayama, M. & Sugihara, R. Which type of washing machine should you choose?: Choosing the type of washing machine. *Int. J. Consum. Stud.* **35**, 237–242 (2011).
34. Niinimäki, K. *et al.* The environmental price of fast fashion. *Nat. Rev. Earth Environ.* **1**, 189–200 (2020).
35. Seyedin, M. Z., Razal, J. M., Innis, P. C. & Wallace, G. G. Strain-Responsive Polyurethane/PEDOT:PSS Elastomeric Composite Fibers with High Electrical Conductivity. *Adv. Funct. Mater.* **24**, 2957–2966 (2014).
36. Ding, Y., Xu, W., Wang, W., Fong, H. & Zhu, Z. Scalable and Facile Preparation of Highly Stretchable Electrospun PEDOT:PSS@PU Fibrous Nonwovens toward Wearable Conductive Textile Applications. *ACS Appl. Mater. Interfaces* **9**, 30014–30023 (2017).
37. Song, J. *et al.* High-Conductivity, Flexible and Transparent PEDOT:PSS Electrodes for High Performance Semi-Transparent Supercapacitors. *Polymers* **12**, 450 (2020).
38. Zhuang, A. *et al.* One-Step Approach to Prepare Transparent Conductive Regenerated Silk Fibroin/PEDOT:PSS Films for Electroactive Cell Culture. *ACS Appl. Mater. Interfaces* **14**, 123–137 (2022).
39. Imura, Y., Hogan, R. M. C. & Jaffe, M. Dry spinning of synthetic polymer fibers. in *Advances in Filament Yarn Spinning of Textiles and Polymers* 187–202 (Elsevier, 2014). doi:10.1533/9780857099174.2.187.
40. Benesty, J., Chen, J., Huang, Y. & Cohen, I. Pearson Correlation Coefficient. in *Noise Reduction in Speech Processing* vol. 2 1–4 (Springer Berlin Heidelberg, 2009).
41. Xu, X., Luo, M., He, P., Guo, X. & Yang, J. Screen printed graphene electrodes on textile for wearable electrocardiogram monitoring. *Appl. Phys. A* **125**, 714 (2019).
42. Qin, Q. *et al.* Electrocardiogram of a Silver Nanowire Based Dry Electrode: Quantitative Comparison With the Standard Ag/AgCl Gel Electrode. *IEEE Access* **7**, 20789–20800 (2019).
43. Reyes, B. A., Posada-Quintero, H. F., Bales, J. R. & Chon, K. H. Performance evaluation of carbon black based electrodes for underwater ECG monitoring. in *2014 36th Annual International Conference of the IEEE Engineering in Medicine and Biology Society* 1691–1694 (IEEE, 2014). doi:10.1109/EMBC.2014.6943932.
44. Correlation coefficients - MATLAB corrcoef - MathWorks United Kingdom. https://uk.mathworks.com/help/matlab/ref/corrcoef.html.
45. Yang, G. *et al.* Adhesive and Hydrophobic Bilayer Hydrogel Enabled On-Skin Biosensors for High-Fidelity Classification of Human Emotion. *Adv. Funct. Mater.* **32**, 2200457 (2022).
46. Xu, Y. *et al.* Multiscale porous elastomer substrates for multifunctional on-skin electronics with passive-cooling capabilities. *Proc. Natl. Acad. Sci.* **117**, 205–213 (2020).
47. Huang, J. *et al.* Tuning the Rigidity of Silk Fibroin for the Transfer of Highly Stretchable Electronics. *Adv. Funct. Mater.* **30**, 2001518 (2020).
48. Pan, L. *et al.* A Compliant Ionic Adhesive Electrode with Ultralow Bioelectronic Impedance. *Adv. Mater.* **32**, 2003723 (2020).

49. SML-811DTT86(A) Data Sheet. https://www.mouser.co.uk/datasheet/2/348/rohm%20semiconductor_sml-81-1201831.pdf (2023).
50. Vaicekauskaite, J., Mazurek, P., Vudayagiri, S. & Skov, A. L. Mapping the mechanical and electrical properties of commercial silicone elastomer formulations for stretchable transducers. *J. Mater. Chem. C* **8**, 1273–1279 (2020).
51. Heo, S., Kim, C., Kim, T. & Park, H. Human-Palm-Inspired Artificial Skin Material Enhances Operational Functionality of Hand Manipulation. *Adv. Funct. Mater.* **30**, 2002360 (2020).
