# Supplementary material for: Imperceptible augmentation of living systems with organic bioelectronic fibres
Source: Nat Electron. 2024 May 24;7(7):586–97. doi: 10.1038/s41928-024-01174-4 (PMC11286532; doi:10.1038/s41928-024-01174-4)
Supplement: Supplementary file 2 — Reporting Summary [file 41928_2024_1174_MOESM2_ESM.pdf]

Corresponding author(s): Yan Yan Shery Huang

Last updated by author(s): Apr 23, 2024

Reporting Summary

Nature Portfolio wishes to improve the reproducibility of the work that we publish. This form provides structure for consistency and transparency in reporting. For further information on Nature Portfolio policies, see our Editorial Policies and the Editorial Policy Checklist.

Statistics

For all statistical analyses, confirm that the following items are present in the figure legend, table legend, main text, or Methods section.

n/a	Confirmed	
☐	☒	The exact sample size (*n*) for each experimental group/condition, given as a discrete number and unit of measurement
☐	☒	A statement on whether measurements were taken from distinct samples or whether the same sample was measured repeatedly
☒	☐	The statistical test(s) used AND whether they are one- or two-sided<br>*Only common tests should be described solely by name; describe more complex techniques in the Methods section.*
☒	☐	A description of all covariates tested
☒	☐	A description of any assumptions or corrections, such as tests of normality and adjustment for multiple comparisons
☐	☒	A full description of the statistical parameters including central tendency (e.g. means) or other basic estimates (e.g. regression coefficient) AND variation (e.g. standard deviation) or associated estimates of uncertainty (e.g. confidence intervals)
☐	☒	For null hypothesis testing, the test statistic (e.g. *F*, *t*, *r*) with confidence intervals, effect sizes, degrees of freedom and *P* value noted<br>*Give P values as exact values whenever suitable.*
☒	☐	For Bayesian analysis, information on the choice of priors and Markov chain Monte Carlo settings
☒	☐	For hierarchical and complex designs, identification of the appropriate level for tests and full reporting of outcomes
☒	☐	Estimates of effect sizes (e.g. Cohen's *d*, Pearson's *r*), indicating how they were calculated

Our web collection on statistics for biologists contains articles on many of the points above.

Software and code

Policy information about availability of computer code

Data collection	All of the data was collected using commercially available software:<br>Electrophysiology signals: RHX data acquisition software version 3.3.1 (updated December 2023).<br>Skin contact impedance: PSTrace v.5 software.<br>Resistance: BV0001B PathWave BenchVue Digital Multimeter App version 2022.<br>Organic electrochemical transistor (OECT): Keithley Kickstart version 2021.<br>Solution rheology: rSpace for Kinexus 2.1<br>Solution surface tension and fibre orientation: ImageJ-win64 2022.
Data analysis	All of the data was analysed with commercially available software: OriginLab (2023 and 2024 versions) and MATLAB R2023a.

For manuscripts utilizing custom algorithms or software that are central to the research but not yet described in published literature, software must be made available to editors and reviewers. We strongly encourage code deposition in a community repository (e.g. GitHub). See the Nature Portfolio guidelines for submitting code & software for further information.

Data

Policy information about availability of data

All manuscripts must include a data availability statement. This statement should provide the following information, where applicable:

- Accession codes, unique identifiers, or web links for publicly available datasets
- A description of any restrictions on data availability
- For clinical datasets or third party data, please ensure that the statement adheres to our policy

All source data (https://zenodo.org/records/10808385) are available in the main text and supplementary information; any additional information needed could be requested from the corresponding author.

Research involving human participants, their data, or biological material

Policy information about studies with human participants or human data. See also policy information about sex, gender (identity/presentation), and sexual orientation and race, ethnicity and racism.

Reporting on sex and gender	Sex and/or gender were not related with this study and were not considered in the study design. Therefore, such information was not collected.
Reporting on race, ethnicity, or other socially relevant groupings	Race, ethnicity, and/or other social relevant groupings were not considered in the study design and such information was not collected.
Population characteristics	8 participants recruited were adult students and staff of University of Cambridge (5 participants for ECG recording and 3 participants for EMG sensing), aged between 18-35, with no known cardiovascular diseases.
Recruitment	Participants were recruited through advertisements within the west technology campus of University of Cambridge. All participants signed up for the experiments are voluntary without pre-selections.
Ethics oversight	Human participant experiments were performed with the approval of the Ethics Committee of the Department of Engineering at the University of Cambridge (7/7/2021, CUEDREC)

Note that full information on the approval of the study protocol must also be provided in the manuscript.

Field-specific reporting

Please select the one below that is the best fit for your research. If you are not sure, read the appropriate sections before making your selection.

☒ Life sciences ☐ Behavioural & social sciences ☐ Ecological, evolutionary & environmental sciences

For a reference copy of the document with all sections, see nature.com/documents/nr-reporting-summary-flat.pdf

Life sciences study design

All studies must disclose on these points even when the disclosure is negative.

Sample size	5 participants were selected for ECG measurements and 3 participants were selected for EMG measurement; for chicken embryo experiments, more than 3 embryos were selected as experimental group (with fibers) and control group (no fiber), respectively, in each round of experiment. No sample size calculation was performed due to the nature of electronics research.
Data exclusions	No data was excluded from the analysis, only except those chicken embryos that were died before fiber printing had been excluded from further experiments.
Replication	For human participants, more than 3 participants were tested for the each experiment and more than 3 repetitions were conducted on each participant, and the results were reproducable. For chicken embryo experiments, 3 rounds of experiments were conducted and the results were repeatable.
Randomization	There is no particular ordering or pre-selection for human participants. Chicken embryos were randomly selected from eggs and allocated in each group.
Blinding	Blinding was not relevant and applicable to this study.

Reporting for specific materials, systems and methods

We require information from authors about some types of materials, experimental systems and methods used in many studies. Here, indicate whether each material, system or method listed is relevant to your study. If you are not sure if a list item applies to your research, read the appropriate section before selecting a response.

Materials & experimental systems

n/a	Involved in the study
☒	☐ Antibodies
☒	☐ Eukaryotic cell lines
☒	☐ Palaeontology and archaeology
☐	☒ Animals and other organisms
☒	☐ Clinical data
☒	☐ Dual use research of concern
☐	☒ Plants

Methods

n/a	Involved in the study
☒	☐ ChIP-seq
☒	☐ Flow cytometry
☒	☐ MRI-based neuroimaging

Animals and other research organisms

Policy information about studies involving animals; ARRIVE guidelines recommended for reporting animal research, and Sex and Gender in Research

Laboratory animals	This study used early-stage chicken embryos (day-2 to day-3) that were extracted from wild type fertilized chicken eggs, acquired from MedEgg Inc. Eggs, UK.
Wild animals	This study did not involve wild animals.
Reporting on sex	Sex was not collected and not relevant to this study because chicken embryos were only used in early-stage status (day-2 to day-3).
Field-collected samples	This study did not involve field-collected animals and other organisms.
Ethics oversight	No animal protocol was required for the chicken embryonic stages studied (< 2 weeks) under the UK Animals (Scientific Procedures) Act 1986.

Note that full information on the approval of the study protocol must also be provided in the manuscript.

Dual use research of concern

Policy information about dual use research of concern

Hazards

Could the accidental, deliberate or reckless misuse of agents or technologies generated in the work, or the application of information presented in the manuscript, pose a threat to:

No	Yes
☒	☐ Public health
☒	☐ National security
☒	☐ Crops and/or livestock
☒	☐ Ecosystems
☒	☐ Any other significant area

Experiments of concern

Does the work involve any of these experiments of concern:

No	Yes	
☒	☐	Demonstrate how to render a vaccine ineffective
☒	☐	Confer resistance to therapeutically useful antibiotics or antiviral agents
☒	☐	Enhance the virulence of a pathogen or render a nonpathogen virulent
☒	☐	Increase transmissibility of a pathogen
☒	☐	Alter the host range of a pathogen
☒	☐	Enable evasion of diagnostic/detection modalities
☒	☐	Enable the weaponization of a biological agent or toxin
☒	☐	Any other potentially harmful combination of experiments and agents

Plants

Seed stocks	Mimosa Pudica and Orchid were used in this work as substrate for fibre deposition demonstration. These plants were purchased from local supermarkets in Cambridge, UK. Dandelions were used as demonstrations for fiber deposition, and they were collected from a grass field, in April 2022 from Cambridge, UK.
Novel plant genotypes	Not applicable.
Authentication	Not applicable.
